# Modification and Recycling Complexity of the Bitumen Modified with SBS Polymer—A Review

**DOI:** 10.3390/ma19183824

**Published:** 2026-09-08

**Authors:** Joanna Szołtysik, Wojciech Sorociak, Sławomir Kwiecień, Jarosław Rybak

**Affiliations:** 1 Faculty of Civil Engineering, Silesian University of Technology, 44-100 Gliwice, Poland; joanna.szoltysik@polsl.pl (J.S.); wojciech.sorociak@polsl.pl (W.S.); slawomir.kwiecien@polsl.pl (S.K.); 2 Department of Civil Engineering, Wroclaw University of Environmental and Life Sciences, 50-375 Wrocław, Poland

**Keywords:** SBS polymer, bitumen, modification, Hot Mix Asphalt, ageing, recycling

## Abstract

This paper presents a comprehensive review of bituminous binder modification using Styrene–Butadiene–Styrene (SBS) copolymer. Bitumen is one of the most widely used binder types in road engineering, and its modification has been extensively studied to enhance performance characteristics. The review outlines the historical development of bitumen modification, discusses binder structural models, and examines the influence of SBS on binder properties. The review focuses on ageing process of Polymer-Modified Bitumen (PMB) and Highly Modified Bitumen (HiMA). The complexity of binder’s origin, polymer structure, ageing factors and the presence of cross-linking agents or other compounds creates significant challenges in predicting binder performance after technological and exploitation ageing. This uncertainty directly affects the assessment of long-term durability and recycling potential. Therefore, it is relevant to incorporate a broader range of ageing factors into standardized laboratory testing procedures to develop effective rejuvenation strategies for SBS-modified PMBs.

## 1. Introduction

**Figure 1:**
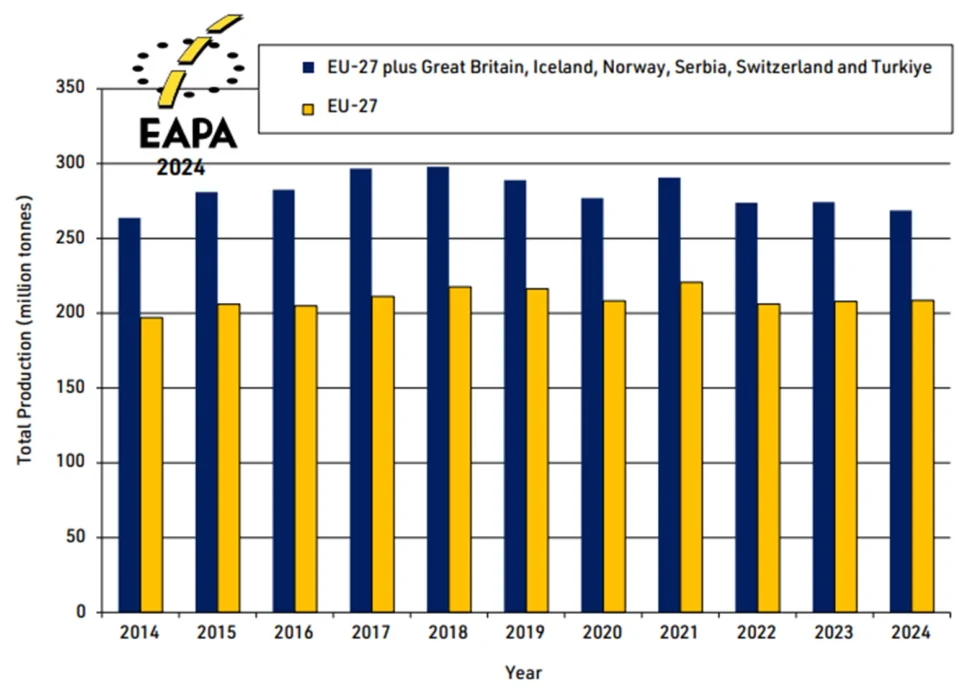
Total production of asphalt in EU-27 and EU-27 plus Great Britain, Iceland, Norway, Serbia, Switzerland and Turkiye, from 2014 to 2024 [4].

Hot Mix Asphalt (HMA) is one of the most popular building materials used in road engineering around the world. As transportation infrastructure significantly affects the global economy, it is crucial to maintain it at certain levels and to search for solutions that are more ecological, durable and financially affordable. According to the National Asphalt Pavement Association (NAPA), in 2011, the production of new asphalt pavement in the US alone was estimated at 500 million tons [1], and based on European Asphalt Pavement Association (EAPA) data, the average annual production in Europe between 2007 and 2013 exceeded 307 million tons [2]. In 2011, the Asphalt Institute and Eurobitume pointed out that the global use of bitumen reaches nearly 102 million tons per year, of which 85% was used for road pavements [3]. After 2011, the production of these materials in Europe decreased slightly and remained stable at 250–300 million tons per year [4] (Figure 1). According to EAPA, for comparison, in the United States, the production of mixtures each year oscillates between 300 and 400 million tons, although NAPA data indicate that these values are underestimated (for 2021 alone, it was 392 million tons according to EAPA [4] and as much as 432 million tons according to NAPA [5], which is 10% more). Most of the binder used in road engineering is refined from petroleum crude oil, and it has been observed that over the years its sources have shrunk and material costs have increased [6].

In recent years, traffic load has increased significantly [7] due to technological development and continuously improving quality of life in many counties within Europe, North America and Asia. For almost a century, the aim of road engineering has been to find ways to improve road materials in terms of fatigue life, low and high temperature resistance, and stiffness. Although HMA consists of approximately 95% aggregates and 5% bitumen, it is the bitumen that is primarily responsible for thermal and rheological properties, as well as ageing processes [8,9,10]. This has led the field of road engineering to focus on binder properties and the use of various kinds of modifiers [3,11] to improve durability, anti-ageing properties [12], the viscoelastic range [13], resistance to permanent deformation [14] and other properties affecting the pavement’s performance.

The aim of this paper is to present the factors, parameters and chemical processes leading to SBS-modified binder ageing and its severity, particularly under high temperatures. To expand the current knowledge about material characteristics, this review encompasses studies on both asphalt binders and aggregate-binder mixtures. This comprehensive scope allows for an assessment of current laboratory ageing methods and their representativeness regarding real-life pavement ageing. Furthermore, it provides deeper insight into the asphalt mixture production process and its subsequent influence on long-term performance and recycling.

### 1.1. Methodology

To collect the relevant literature for this review, a systematic search was conducted using the Google Scholar database. The search strategy utilized a tiered combination of keywords. Initial screening involved general terms such as “SBS modified binder”, “PMB ageing” and “reclaimed asphalt pavement”. Following this, the search was narrowed down by separating “PMB” and “HiMA” binders, and subsequently applying specific keywords regarding degradation mechanisms, including “cross-linking”, “polymer degradation”, “UV ageing” and “mixing plant”. The inclusion criteria targeted studies focusing on the factors, chemical processes and high-temperature parameters leading to the ageing of both asphalt binders and aggregate-binder mixtures. The literature was strictly limited to peer-reviewed papers published mostly in English and Polish, with addition of standardized statutes. There was no limitation to the search period. The primary exclusion criterion was the focus on other binder modifiers, which were omitted from this analysis except for sporadic comparative purposes. A final selection of relevant studies was then synthesized using a bottom-up approach, transitioning from molecular and chemical parameters to large-scale asphalt mixture production.

Additional statistical analysis of reviewed data was performed using R Studio ver. 2026.06.0+242 based on R 4.6.1 environment. Statistical analysis contains mostly Pearson correlation test with significance t-test addition and ANCOVA for comparison of various mathematical models. Software was also used to recreate graphical representation of original data and comparative heatmaps.

### 1.2. History of Binder Modification

The first technology for bitumen modification was patented in the mid-19th century [15,16] and involved both natural and synthetic polymers. In the 20th century, the global use of modified binders developed both in US and Europe [15,17,18,19]. In the late 1970s, the production of modified binders was much higher in Europe than in the US [17]. One probable reason for this phenomenon was higher initial costs of that technology in the US. Development in Europe was also associated with higher motivations to limit the costs of exploitation of the pavement and expand its life cycle [18,19]. At the turn of the century, the US expanded its polymer-modified bitumen (PMB) industry, including surveys conducted for state departments of transportation [20] and research evaluating the influence of polymer modification on pavement properties [21,22,23,24]. All of this occurred as a consequence of the establishment of SHRP (Strategic Highway Research Program) in 1987, when rheology tests gained a major role in the evaluation of binder properties [25], and the Superpave protocol was created. During that time, various types of polymers were investigated globally as binder modifiers.

Polymer modifiers are divided into plastomeric and elastomeric types [26]. The main difference between them is their susceptibility to reversible deformation. Plastomers retain their shape at room temperature and exhibit negligible reversible elasticity, which leads to degradation under high loads [27]. Originally, they can be divided into thermoplastics with linear structure, which can be re-molded at high temperatures by weakening the intermolecular forces and thermosets with a covalent, cross-linked structure formed at high temperatures, which is non-reversible. Elastomers are polymers that can be subjected to stress with a high strain response; however, they almost completely return to their original shape. These properties are caused by polymer chains randomly connected with cross-links, and this irregularity prevents the elastomer from developing a crystalline, irreversible structure [28].

The polymers most frequently used in bitumen modification are plastomers and thermoplastic elastomers [27]. Thermoplastic elastomers are copolymers combining elastomeric and plastomeric components, such as Styrene–Butadiene–Styrene copolymer (SBS). SBS is the most widely used polymer in the transportation industry [29].

## 2. SBS-Modified Bitumen

### 2.1. SBS Polymer

**Figure 2:**
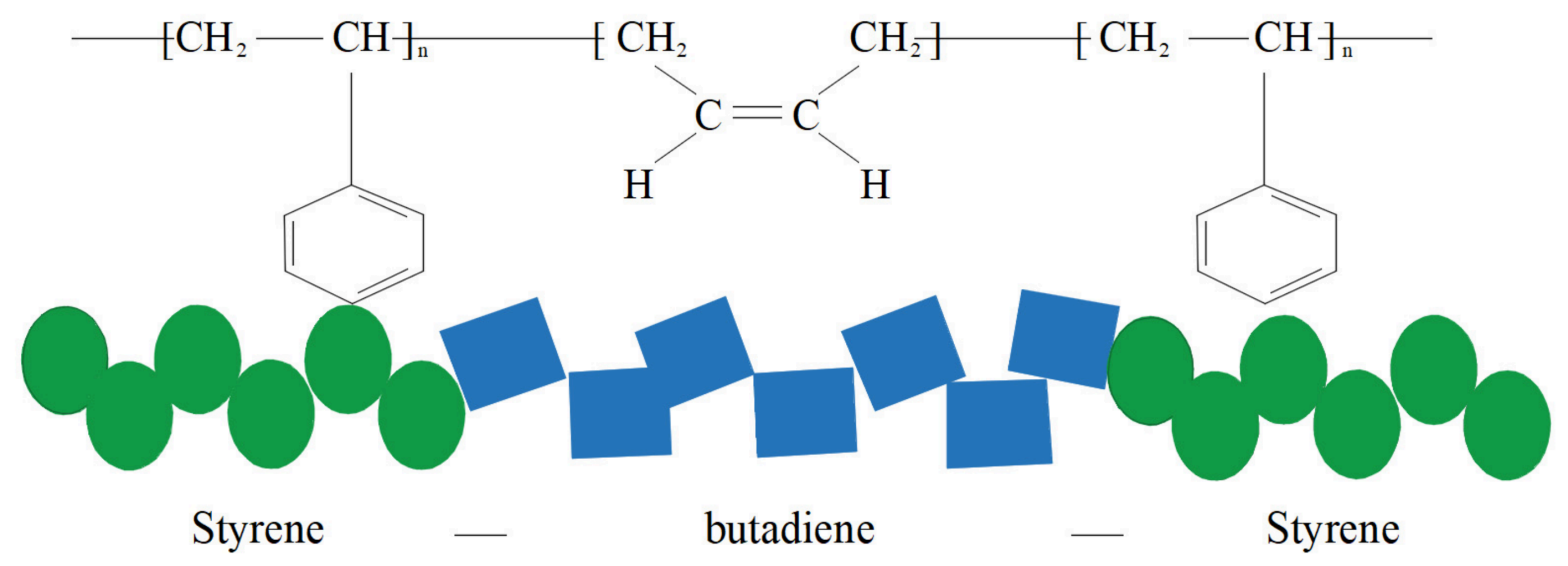
Structure of SBS polymer, reprinted from [30] under the terms of open access (CC-BY 4.0 license).

**Figure 3:**
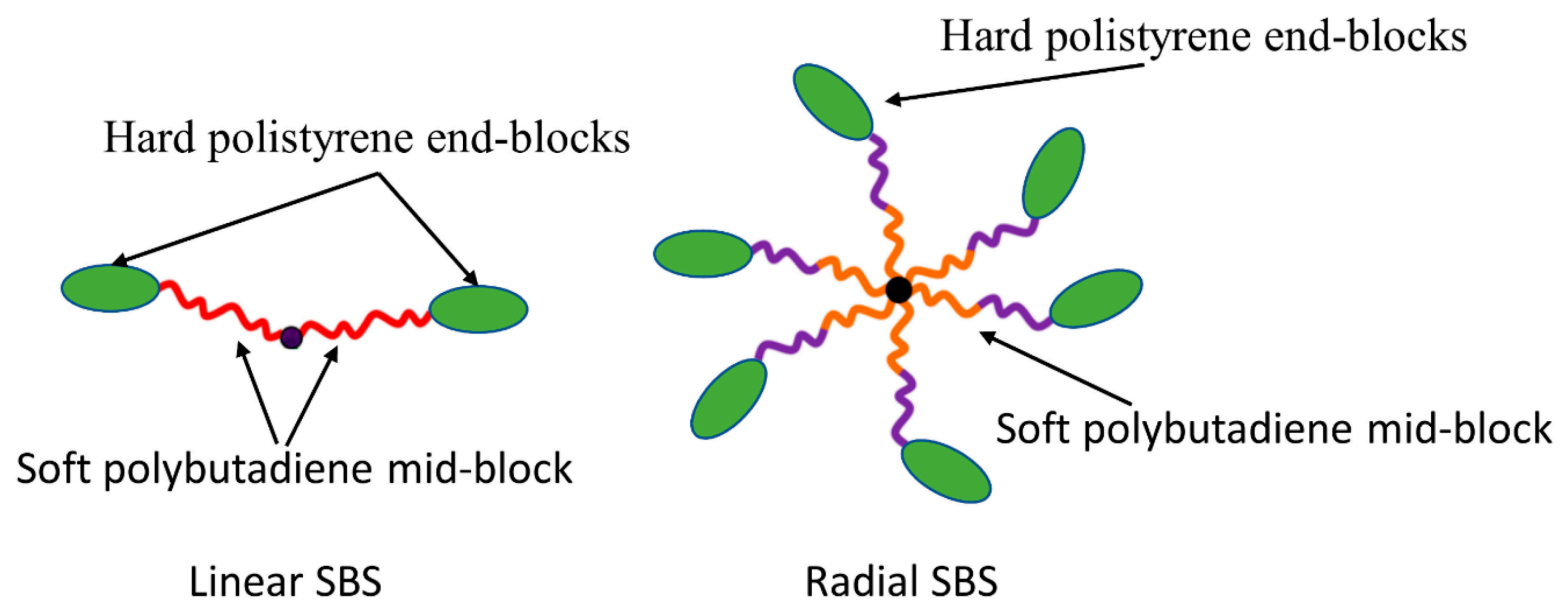
Linear and radial SBS polymer reprinted from [36] under the terms of open access (CC-BY 4.0 license).

SBS copolymer is a three-block polymer that consists of two block types—stiff polystyrene and elastic polybutadiene (Figure 2) [30]. Although the SBS polymer itself is susceptible to ageing, particularly in terms of photo-oxidation resistance, which is considered to be very low for both polystyrene and polybutadiene [31], the combined effect of binder modification with SBS shows some ageing resistance of the polymer caused by the presence of maltenes [32]. The modified binder and asphalt pavement also achieve better performance in terms of fatigue, rutting and ageing resistance while remaining flexible enough to be more resistant to low-temperature cracking [33]. There are two types of SBS polymer structure—linear and radial [34,35,36] (Figure 3), which also have influence on binder properties, as discussed in the following chapters of the paper. The main disadvantages of SBS polymer, namely low storage stability and polymer degradation, can cause difficulties in compaction of asphalt mixture and recycling. These problems are caused by the types of chemical bonds forming the polymer structure and their interactions with the environment and the binder. An uncontrolled increase in viscosity is also considered as disadvantage in terms of asphalt mix production and compaction.

Usually, polymers are molecules held by covalent bonds and connected with each other or to separate molecules by intermolecular (secondary) forces (for example, van der Waals forces) [37]. Through oxidative and thermal reactions, irradiation and the presence of other compounds, double covalent carbon bonds are susceptible to chain scission and cross-linking [38,39,40]. These chemical reactions cause changes in SBS structure during the production and exploitation of the asphalt mixture and influence its properties over time.

### 2.2. Binder Chemical Structure

Bituminous binder is a viscoelastic material consisting of different hydrocarbons. Their content depends on the binder’s origin and affects the properties of the material. Since the mid XIXth century, many methods of binder separation have been developed, and the fractions of bituminous binder are now distinguished as [41,42,43,44,45]:

- saturates,
- aromatics,
- resins,
- asphaltenes.

Gaestel introduced the Colloidal Index IC (1), noting flocculation between part of the maltenes and asphaltenes and combining them into larger groups in colloidal model [43,46]. The binder colloidal instability described by Gaestel was influenced by the presence of flocculants, which affected asphaltene isolation, in contrast to fractions acting as peptizing agents, which dispersed the asphaltenes and prevented their separation. Therefore, IC is presented as ratio of the sum of asphaltenes and flocculants to the surfactants [43]. Since flocculants are a mixture of saturates and aromatics, and surfactants the mix of the resins and aromatics, the simplified IC (1) can be calculated using the following weight ratio [47]:

(1) $$I_{c}=\frac{Asphaltenes\;+\;Saturates}{Resins\;+\;Aromatics},$$

The viscoelastic behavior of bituminous binder is related to its range of hydrocarbon fractions. The viscoelastic range of bitumen plays a significant role in transportation industry, particularly in temperate climate regions, where air temperatures vary throughout the year and reach both high and low extremes. Such temperature variations can lead to pavement damage, including rutting and thermal cracking [48,49]. Moreover, the viscoelastic properties of bitumen change over time [50] due to oxidation and thermal ageing, which cause alterations in binder fraction content [51]. This brief explanation highlights the importance of viscosity and colloidal properties in modified binders, where polymers significantly influence segregation and phase separation.

The first tests developed to determine the performance properties of bitumen were the penetration [52], softening point [53], and Fraass breaking point tests [54], which are still performed today. In Europe, these tests are still used to classify bitumen types and assess their suitability for high or low temperature environments. The penetration index (PI), an indicator of temperature sensitivity, is also determined as classification method.

The penetration test is direct physical determinant of the binder viscosity and can be used to evaluate the storage stability of bitumen. Binders with lower viscosity exhibit much higher penetration values compared to the high-viscosity binders under the same temperature. This parameter is strongly correlated with softening point, which defines the characteristic temperature at which bitumen transitions from a solid to a non-Newtonian liquid [55,56]. A correlation also exists between penetration, softening point and SARA fractions content, including IC [42].

The second characteristic temperature describing the binder behavior is Fraass breaking point. Both softening and breaking point are mutually dependent and increase with higher asphaltene content [57]. Observing their correlative behavior indicates that the viscoelastic range of binder is limited, although characteristic temperature of individual binders depends on their origin. Therefore, it is challenging to find asphalt binders that exhibit proper viscoelastic behavior across a wide temperature range.

Since all of these parameters tend to change over time due to oxidation, mechanical and thermal influences, and other chemical reactions, the raw asphalt binder and asphalt mixtures are very sensitive to ageing and undergo significant changes in their properties during their lifetime. This became the starting point for finding ways to improve the viscoelastic range of bitumen, as well as its strength and fatigue resistance, with a focus on rheological parameters and modifiers.

### 2.3. Microstructural Properties of PMB

The interaction of the SBS polymer with bitumen is a combined effect of the compatibility of the polymer blocks with each bitumen fraction. Considering the linear viscoelastic model, the binder is compared to a low-molar-mass polymer [58]. Some sources describe the chemical components of bitumen as oligomers [33,59] that interact with the SBS polymer structure depending on the particular fraction. It has been shown that asphaltenes do not interact significantly with the polymer blocks, and the mixture of these components does not change the glass transition temperature of the asphaltenes. On the contrary, the maltene/polymer mixture does not separate; instead, the components interact with each other [59] due to the absorption of some of the light fractions and the swelling of the polymer. Separation also occurs between the polymer blocks, since the polybutadiene and polystyrene blocks are not compatible. As a result, the polystyrene phase, with a much higher glass transition temperature, disperses within the elastic matrix of polybutadiene [13,60].

One of the main indicators of PMB performance is the dispersion of the SBS polymer in the asphalt binder [61]. The dispersion is characterized by the term ‘morphology’ and is observed using fluorescence microscopy [60,62,63,64] and Atomic Force Microscopy (AFM), which overcomes the resolution limitations and scale constrains of fluorescence microscope [65]. The morphology of PMB is affected by a polymer content of approximately 3–7% by weight [13], the content of asphaltene and maltene fractions, and the ratio of polystyrene to polybutadiene in the SBS structure [61].

**Figure 4:**
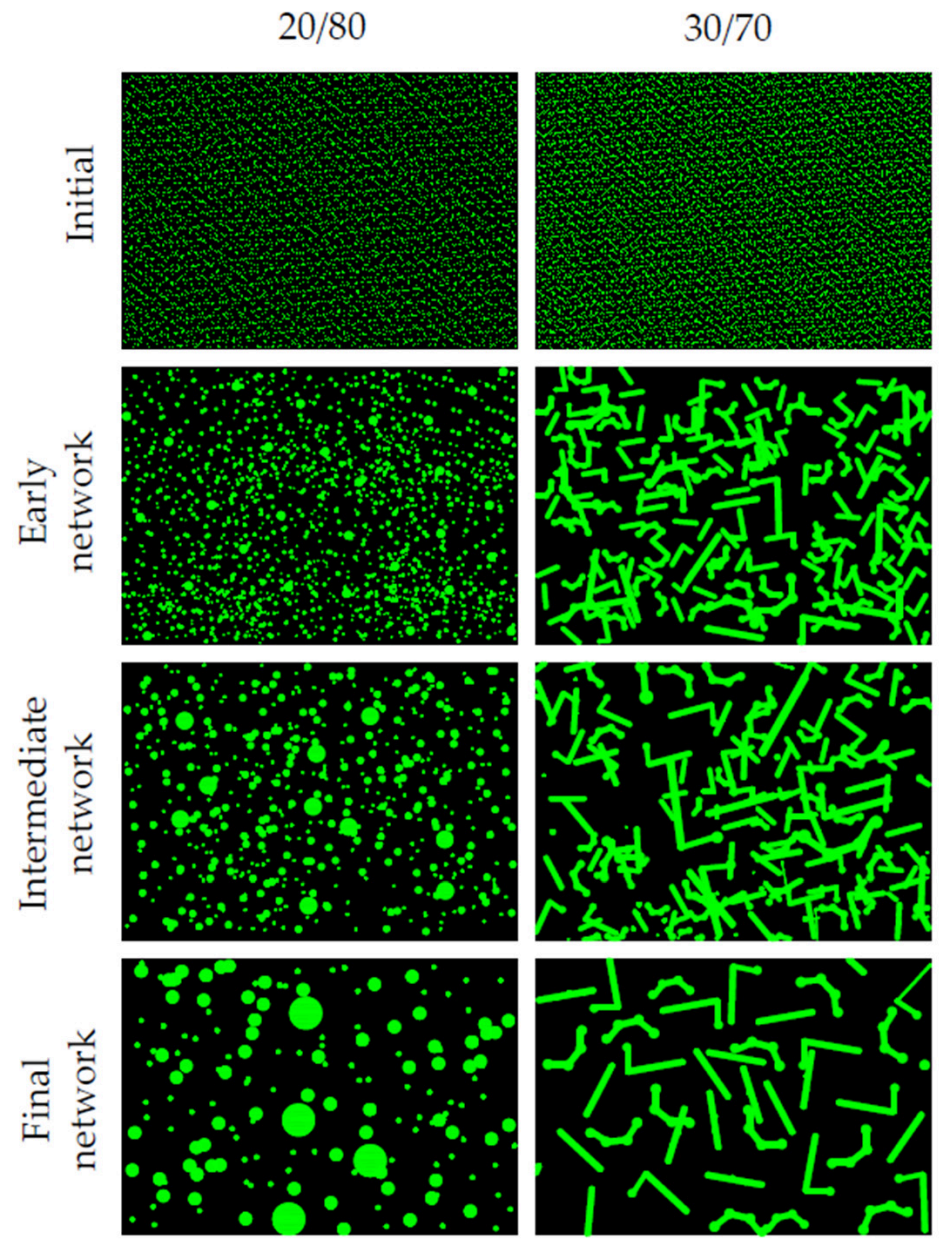
Graphic diagram of morphology evolution of the SBS polymer under 130 °C with S/B ratio 30/70 and 20/80, based on [66].

The S/B ratio affects the morphology of PMB due to intermolecular forces between the PS blocks [29]. A lower content of PS blocks within the SBS structure results in weaker intermolecular forces, which leads to better dispersion of the polymer in the PMB structure (Figure 4) [66]. The dispersion of the polymer blocks prevents phase separation of the binder. Moreover, the radial shape of the polymer results in a more irregular dispersion in the asphalt binder than the linear shape, making linear SBS more compatible with the binder [61].

**Figure 5:**
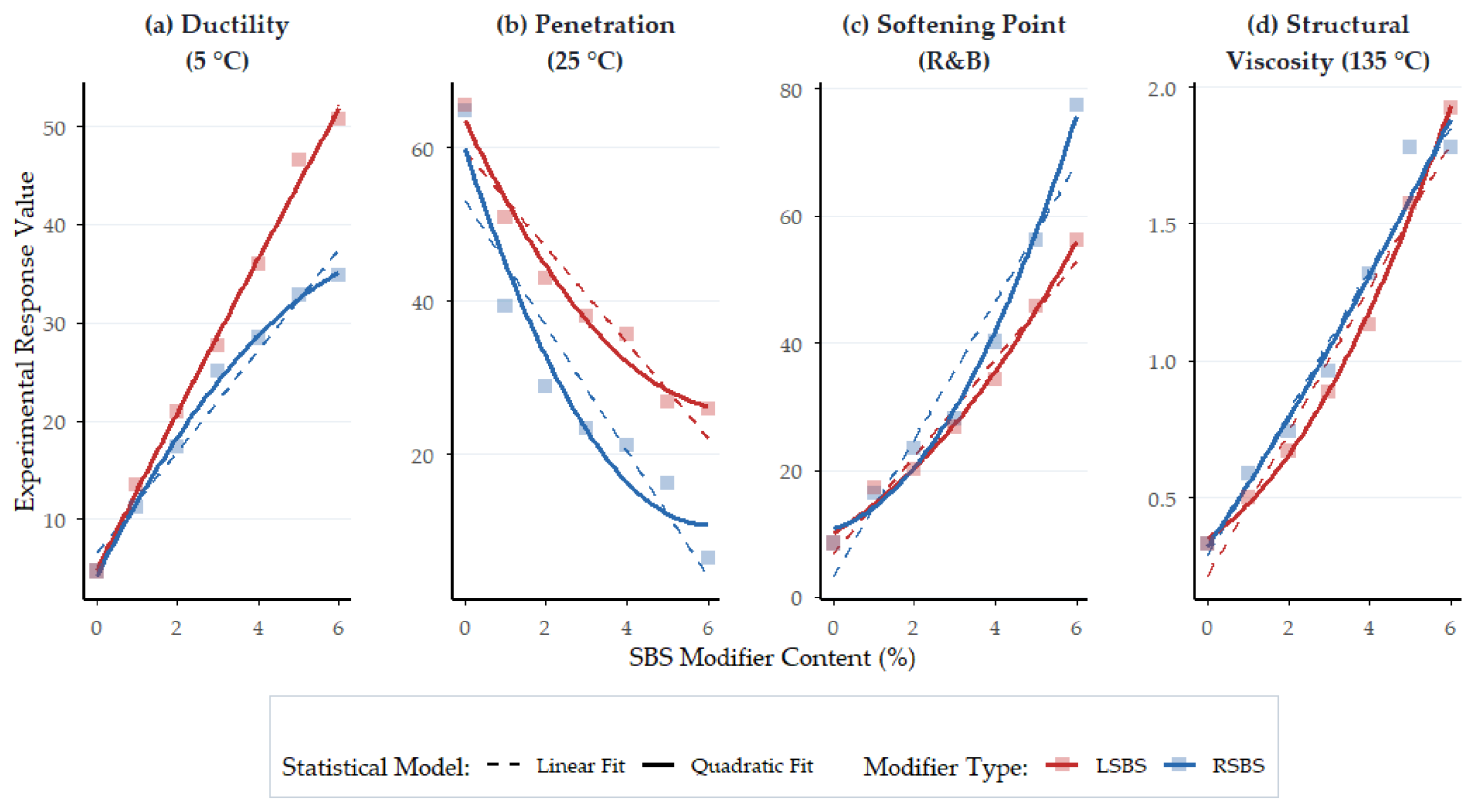
Physical properties of SBS-modified bitumens: (**a**) viscosity, (**b**) softening point, (**c**) penetration and (**d**) ductility with linear and quadratic model, based on [67].

There are two types of SBS polymer structures—linear and radial [34,35,36]—which also influence binder properties. Studies show that the radial structure of the SBS polymer has a greater impact on binder properties, affecting high-temperature resistance, viscosity and rheological properties [67]. Despite identical polystyrene-to-polybutadiene ratios in both copolymers (30/70), differences in the weak bonds within the polymer lead to these changes. Authors, however, used linear model to determine the differences between RSBS and LSBS. To investigate the limitations of original linear regression and evaluate the complex effects of polymer content and modifier type, a comparative ANCOVA framework was established with a significance level α = 0.05. The regression baseline was shifted from a simplified linear model (y = ax + b) to a rheologically justified quadratic model (y = ax^2^ + bx + c) (Figure 5). The structural analysis of the model residuals confirms a profound increase in mathematical accuracy, as evidenced by a drastic reduction in the Residual Sum of Squares (RSS) across all evaluated physical properties. The most prominent error reduction was achieved for the Softening Point, where the RSS dropped from 265.72 (linear) to 42.47 (quadratic), and for Penetration, decreasing from 361.53 to 152.86 (Table 1).

**Figure 6:**
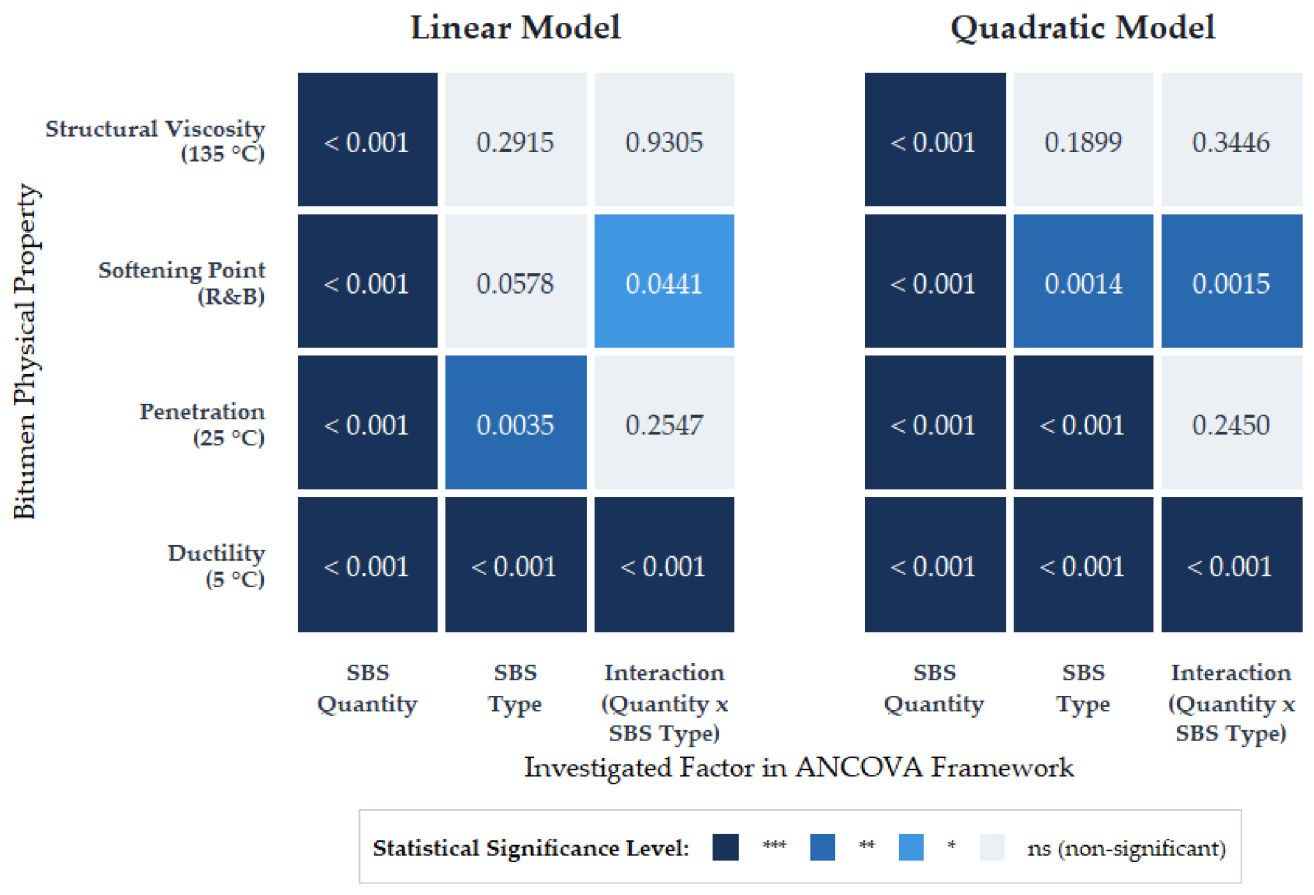
Comparison of statistical significance (*p*-values) between the simplified linear model and the rheologically justified quadratic model within the ANCOVA (***—*p* < 0.001, **—*p* < 0.01, *—*p* < 0.05, ns—non-significant), based on [67].

The statistical outputs demonstrate how the choice of the mathematical model crucially dictates the engineering conclusions. For the Softening Point, while the original linear model showed a weak statistical significance between the RSBS and LSBS (*p* = 0.04412, *p* < 0.05), the quadratic model unveiled a highly significant interaction effect (*p* = 0.00154, *p* < 0.005). This statistically confirms that the RSBS modifier achieves an accelerated, progressive rate of thermal stability enhancement at higher concentrations compared to the LSBS modifier. Also, according to the quadratic model, the starting points of the unmodified binder models is statistically equal (*p* = 0.00137, *p* < 0.005) in comparison to the linear model, where the comparison of the starting point is statistically insignificant (*p* = 0.05782, *p* > 0.05), which is not possible from an engineering point of view (the used base binder is the same material for both ways of modification) (Figure 6).

Conversely, for Penetration at 25 °C, the interaction term between two types of modifiers was found to be statistically non-significant in both linear and quadratic models (*p* = 0.24547 and *p* = 0.24497, respectively, *p* > 0.05). In ANCOVA, a non-significant interaction paired with an extremely significant main effect of the Modifier (*p* = 0.00079 < 0.001 for quadratic model and weaker *p* = 0.00347 > 0.05 for linear) implies a clean vertical shift between two statistically significant trajectories. This reveals that while both polymers restrict bitumen penetration at an identical structural rate (curve of the models is not significantly different), the RSBS matrix maintains a consistently distinct, lower baseline of penetration throughout the entire dosage spectrum, proving its superior stiffening capability.

For low-temperature Ductility (5 °C), the quadratic interaction term reached an extreme level of significance (*p* = 0.00009). This indicates a fundamental divergence in the failure physics of the two binders. The RSBS binder undergoes rapid over-modification at higher concentrations leading to structural embrittlement, whereas the LSBS modifier maintains a stable, continuous ductile progression. Finally, for Structural Viscosity at 135 °C, the non-significant interaction (*p* = 0.3446) under a substantially lower residual error (0.0632 vs. 0.1308) confirms that both modifiers influence the technological flow behavior through an identical non-linear structural mechanism.

The content of SBS in the asphalt binder has the greatest impact on its morphology and properties among the listed conditions. A low content of SBS polymer leads to a colloidal model, in which the binder forms the continuous phase with dispersed polymer particles. Above a 7% content by weight (or, according to some sources, 6% [68]), the maltenes become insufficient to swell the polymer components, and SBS forms a cross-linked matrix with dispersed binder particles [61], resulting in new properties as Highly Modified Bitumen (HiMA) [65]. HiMA exhibits predominantly the properties of the SBS polymer, since it constitutes the majority of the PMB by volume.

The cross-linking of polymers is a process that can occur both physically and chemically [69]. Physical cross-linking is formed by weak, reversible interactions, which are among the main properties of thermoplastic elastomers. It allows the material to be remold at high temperatures, when the kinetic energy of the particles exceeds the intermolecular forces, thereby restoring its properties after cooling [3]. In contrast, chemical cross-linking occurs due to the presence of other compounds, irradiation, heat, or pressure, which create covalent bonds. This process changes the structure of the polymer matrix and reduces the remolding ability of thermoplastic polymers. Considering the swelling of the polymer [70] and the insufficiency of maltenes to dissolve high-molecular-weight fractions in the presence of the polymer, chemical cross-linking can permanently alter the binder’s colloidal system and its properties.

Polymer-modified bitumen represents kinetically stable but thermodynamically unstable system [13]. Thermoplastic elastomers differ from the asphalt binder in terms of molecular structure, density, and polarity, given the variety of hydrocarbon types present in bitumen [71]. However, they have a molecular weight similar to that of asphaltenes, therefore, the solvency of maltenes may become insufficient for both components, leading to phase separation [61]. Moreover, due to polymer swelling, the concentration of asphaltenes in the PMB matrix further increases [43]. Considering the colloidal index IC (1), a higher asphaltene content and a large quantity of light fractions absorbed by the polymer significantly affect binder stability, as asphaltenes cannot be properly dispersed within the aromatic phase and may separate [72]. Storage stability is also correlated with the chemical structure of the SBS polymer, which contains unsaturated C=C double bonds in the polybutadiene structure [73] and may be influenced using cross-linking agents such as sulfur.

One of the indicators of binder stability is the softening point test performed after storage in aluminum foil tube, which is peeled after storage and cut into sections corresponding to the top and bottom parts of the storage container. Differences in softening point results indicate incompatibility between the binder and the polymer, since during separation, the polymer tends to migrate to the top [26]. However, this method has been described as less reliable in the case of modified binders due to differences in viscoelasticity between the binder and the polymer and the uneven influence of temperature during the hot storage test on the top and bottom fractions [74,75]. Fluorescence microscopy visually reveals the morphology of the modified binder during storage; however, it does not detect changes in the chemical structure and therefore cannot directly quantify the polymer content [76,77].

In addition to other methods, various spectroscopy tests can be utilized to evaluate the storage stability and general chemical characteristics of bitumen [78]. However, when discussing spectroscopic techniques, their inherent limitations must be critically addressed. Classical Fourier-Transform Infrared Spectroscopy (FTIR) has been found unreliable for bitumen analysis due to the material’s strong infrared absorbance, which prevents full IR beam penetration (as FTIR often requires completely transparent samples). To overcome this issue, the Attenuated Total Reflectance (ATR-FTIR) method has gained widespread popularity, as the evanescent wave penetrates the sample only by a few micrometers. To ensure reliable results, sample preparation must be performed in the bulk state by preheating the binder and pressing it firmly onto the crystal to achieve a uniform contact surface [79]. Nevertheless, some studies have utilized solvent-casting approaches (usually using dichloromethane) instead of direct solid-state application. This practice can lead to an uneven film on the crystal; furthermore, the volatile solvent may not evaporate completely, remaining entrapped within the dense bituminous matrix and generating severe spectral artifacts [80]. Many studies do not describe the sample preparation for the FTIR analysis [81].

It is also important to note that raw ATR-FTIR results for bitumens are inherently semi-quantitative. To accurately quantify the analyzed components, calibration curves—which mathematically define the relationship between a specific spectral feature and a known physical property—are essential. Although the Beer–Lambert Law dictates that absorbance is directly proportional to concentration, raw absorbance or transmittance values alone provide no direct information about the exact mass percentage of a substance [82]. However, establishing a universal calibration curve for asphalt binders in general is practically impossible due to their complex nature as highly variable mixtures of diverse hydrocarbons without a fixed chemical structure. Consequently, to monitor modifications in the binder functional groups, indexes for each important substance are widely implemented. The index of any given functional group is calculated as the ratio of the area under its characteristic peak to a stable reference peak area. This mathematical approach successfully quantifies the relative percentage growth or decreases of functional groups after chemical modification or conditioning, rather than their absolute mass content [83].

The Dynamic Shear Rheometer (DSR) and Dynamical Mechanical Analysis (DMA) are also used as indicators of storage stability through rheological properties [76], which show a strong correlation with spectroscopic results (the average coefficient of determination R2 between wavelength differences describing the SBS content and the Separation Index obtained from DMA results is 0.9, according to [74]).

### 2.4. Physical and Rheological Properties of PMB and HiMA

The benefits of binder modification are mainly related to rheological properties and resistance to ageing, oxidation, fatigue cracking, and other failures strictly associated with the viscoelastic behavior of the binder [13]. Although the SBS polymer exhibits susceptibility to thermal, atmospheric and photo-oxidation [84], PMB demonstrates better ageing resistance than the unmodified binder due to the phenomenon of absorption and swelling of light fractions of the bitumen by the polymer network [85].

Research conducted between 2018 and 2020 shows that, for bitumen with similar penetration values but different contents of the SBS modifier, the softening point test results change significantly (Table 2) [86]. These findings indicate that asphalt binders with similar penetration at room temperature, but higher polymer contents exhibit better resistance to heat and rutting.

The extension of the viscoelastic range of the binder through modification mainly occurs by increasing the softening point. Low-temperature resistance, determined by the Fraass breaking point, is also improved, but not that significantly—the average difference between raw binder and HiMA for bitumen produced in 2018–2020 is 6.5 °C [86].

Rheology tests have gained an important role in binder research because the binder undergoes viscoelastic deformations; it does not fully return to its original after stress application, and those deformations tend to accumulate. Due to the polymer network in PMB, the viscosity of the binder is much higher in comparison to the raw binder. Moreover, PMB viscosity is significantly higher compared to that of the unmodified bitumen with increasing temperature. This necessitates higher production and transport temperatures for the modified binder in order to achieve the viscosity required for proper compaction and density of the asphalt pavement. However, elevated temperatures accelerate the oxidation process of the binder and may affect the cross-linked polymer structure (particularly in the case of HiMA), which can catalyze ageing process and damage the initial polymer structure. The elasticity provided by polymer modification is evaluated by ductility and elastic recovery tests. The average elastic recovery of modified binders within the 45/80 penetration range before RTFOT testing exceeds 95% for HiMA 45/80-80 and 85% for PMB 45/80-55, compared to the required values 80% and 70%, respectively [86]. The high elastic recovery of HiMA results from polymer cross-linking and its volumetric predominance in the asphalt binder.

**Figure 7:**
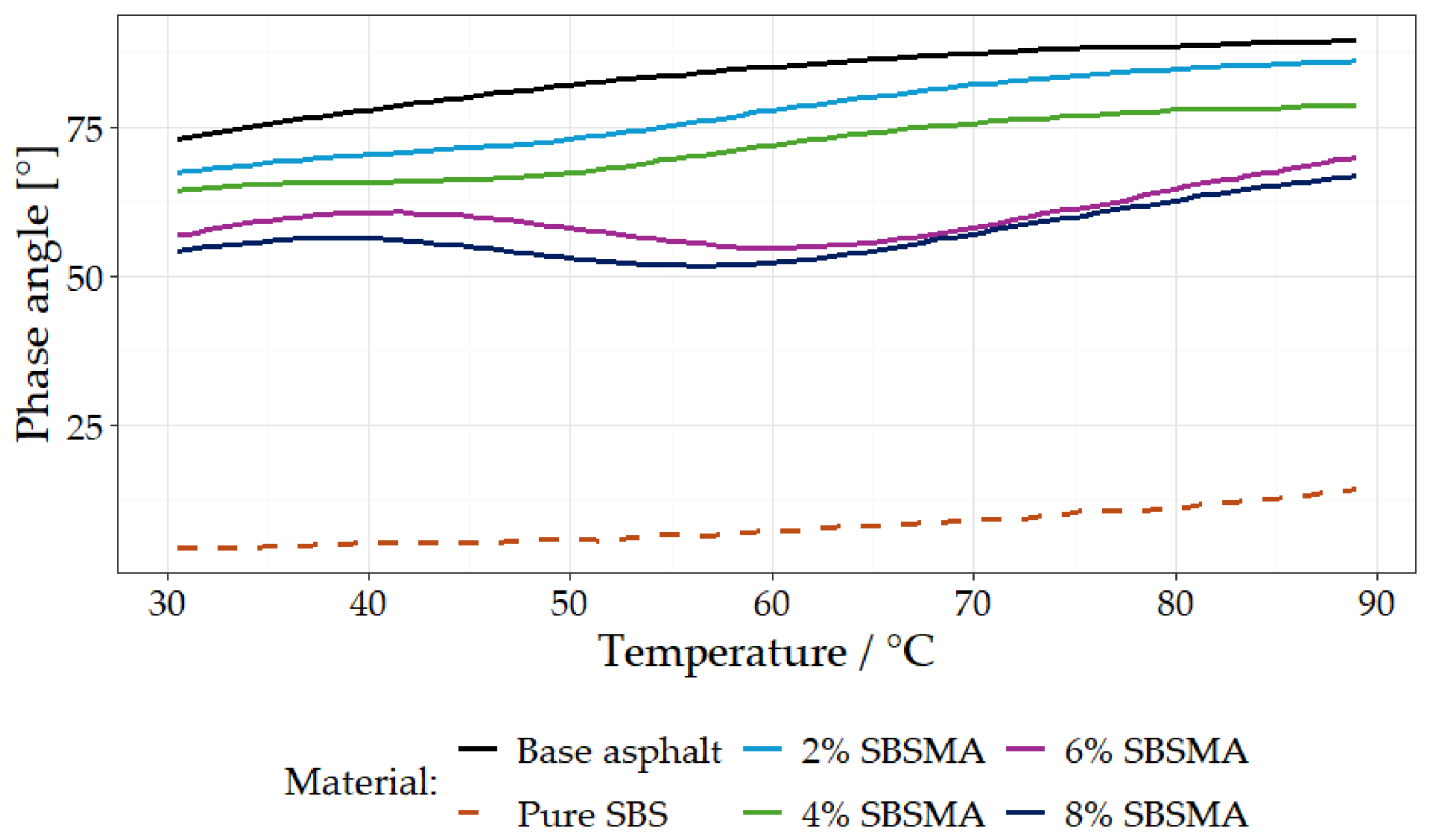
The rheological behaviors of PMB with different SBS dosages and SBS modifier, bitumen matrix and PMB, based on data from [88].

Rheological tests for asphalt binders are performed using a Dynamic Shear Rheometer (DSR). The standard procedure provides the complex shear modulus (G*), which consists of both elastic and viscous components, as the binder is a viscoelastic material, as well as the phase angle (δ). The Superpave system introduced the rutting parameter (G*/sin(δ)), which was one of the first rheological indicators of bitumen susceptibility to rutting [87]. A comparison of the phase angles of binders with different SBS contents reveals distinct PMB behavior within specific temperature ranges, with lower phase angle values observed for binders containing higher amounts of the modifier (Figure 7) [88], where the roles of the asphalt binder and polymer dispersion are reversed. The authors explain the ‘plateau’ phenomenon by the dominance of the polymer structure over the rheological properties of the binder between 30 °C and 60 °C. This effect is more pronounced in binders with higher SBS contents, where cross-linking occurs and increases the elastic recovery of the binder [61,70]. Moreover, the polymer properties alone do not account for the plateau appearance, since no visible nonlinear change in phase angle is observed for raw SBS (Figure 7) [88]. This indicates that it is the interaction between the binder and the modifier that creates the structural complexity of PMB and alters the phase angle behavior.

**Figure 8:**
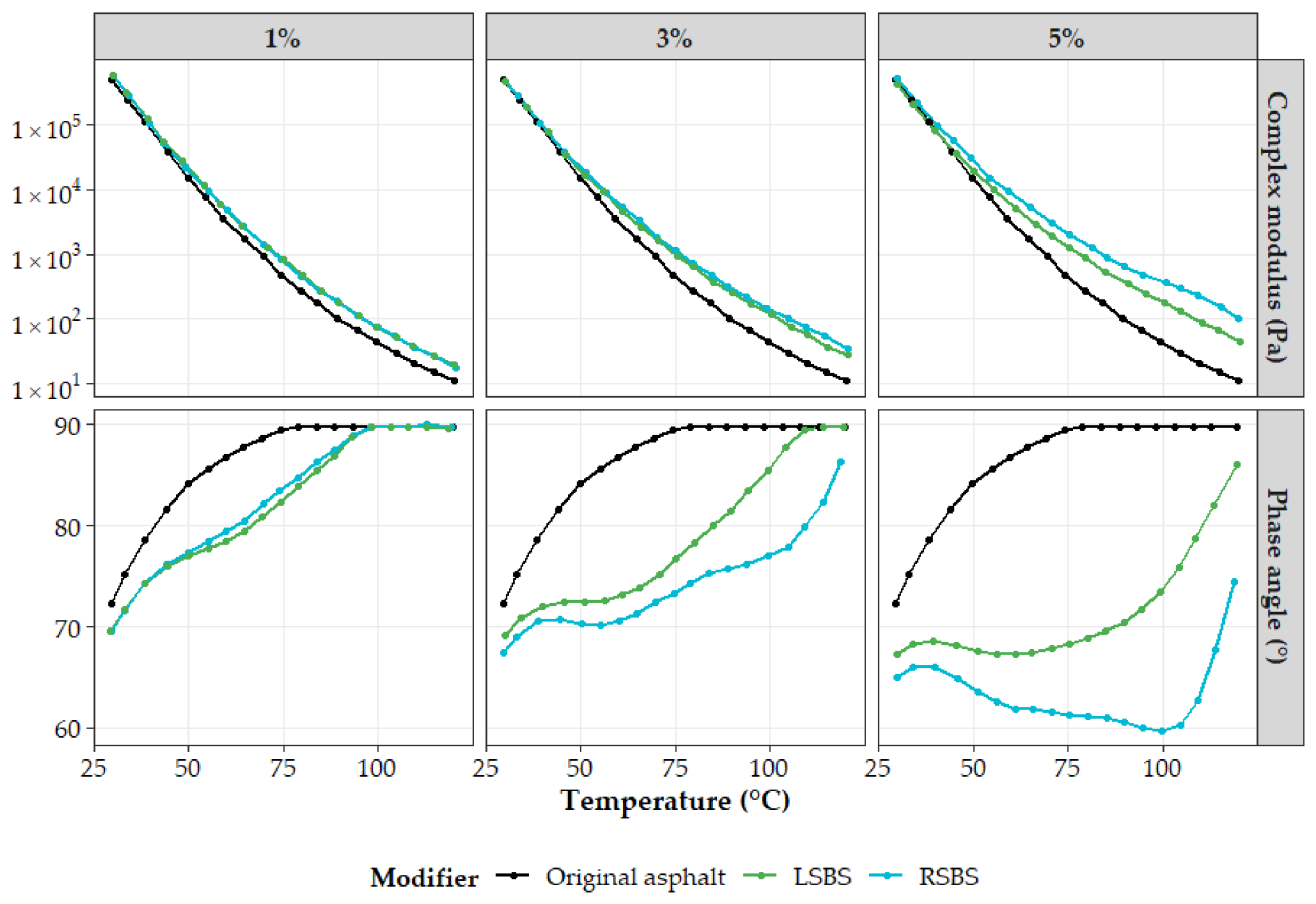
Rheological properties of modified bitumens’ complex stiffness modulus (**top**) and phase angle (**down**), according to [67].

Figure 8 shows results from temperature sweep test in DSR apparatus—complex modulus (G* [Pa]) and phase angle (δ [°]) in a temperature range 30 °C–120 °C for LSBS and RSBS [67]. After adding 5% of SBS polymer (both types), there is a visible plateau region informing about polymer matrix creation (PMB converts to HiMA). To compare the rheological differences between the polymer type and content, the ANCOVA test was performed on log(G*) and log(G*/sin(δ)). The mathematical cubic model for log(G*/sin(δ)) was necessary to evaluate the influence on the phase angle, since the phase angle alone is described by much more complicated curve.

In Table 3, there are results from ANCOVA test for log(G*). The “Intercept” value is a baseline estimate for LSBS content. The number rises with the higher LSBS content; hence, higher LSBS content causes a higher complex modulus. Row “Modifier Type: Unmodified Matrix” is an estimate showing the average vertical difference between the LSBS and unmodified matrix. Negative value means, that complex modulus is lower for the unmodified binder than with LSBS modification. The interesting part is the row “Modifier Type: RSBS polymer”. The first dosage (1%) shows that RSBS causes a slightly lower average complex modulus than LSBS. However, with higher polymer content, RSBS greatly surpasses LSBS in terms of stiffness. “Linear Temperature × [Modification Type]” is a temperature sensitivity parameter (curve slope). In comparison with unmodified bitumen, LSBS is far more resistant to temperature. In case of RSBS, 1% content the difference in temperature sensitivity in comparison to 1% LSBS is statistically non-significant, so their temperature performance is equal. However, after 3% addition, RSBS “flattens” curve slope, making RSBS-modified bitumen more resistant to temperatures.

To evaluate the high-temperature performance and permanent deformation resistance across the tested temperature range, separate cubic ANCOVA models were established for the rutting susceptibility factor (log(G*/sin(δ)), using the LSBS polymer modification as the reference baseline (Table 4). The statistical parameters confirm that the geometric trajectory and global magnitude of the rutting parameter are highly dependent on the modifier type and its concentration. As polymer content increases, the baseline gap for the neat binder (“Modifier Type: Unmodified matrix) broadens significantly, shifting from β = −0.1995 at 1% to a profound gap of β = −0.3934 at 5% content. Simultaneously, a significant modifier threshold effect is observed. At a low dosage of 1%, RSBS exhibits a slightly lower rutting factor than LSBS (β = −0.0132). However, an engineering inversion takes place at higher concentrations, where RSBS establishes a distinct statistical advantage, yielding higher overall values at 3% (β = 0.0679) and reaching a maximum enhancement of β = 0.2447 at 5% polymer content. Similarly to complex modulus test, interaction terms were calculated. While the interaction for RSBS at 1% concentration remains statistically non-significant (*p* > 0.05), indicating a parallel thermal degradation rate to LSBS, the interaction effect becomes highly significant and strongly positive at higher dosages. For 3% and 5% content, the interaction coefficients reach β = 0.2705 and β = 0.7736, respectively. This substantial mitigation of the primary negative temperature slope mathematically proves that RSBS polymer network is progressively more efficient than LSBS at preserving structural integrity and resisting shear deformation during hot-weather exposure.

A complementary ANCOVA model for the phase angle component mathematically mirrors the structural changes observed in rutting parameter, confirming that the superior mechanical resistance of RSBS stems from an enhanced elastic response. At a low dosage of 1%, RSBS exhibits no statistical elasticity difference compared to LSBS (*p* > 0.05). However, matching the critical polymer threshold effect, RSBS introduces a significant negative shift at 3% (β = −0.0060, *p* < 0.001) and reaches its maximum elastic superiority at 5% content (β = −0.0241, *p* < 0.001). Furthermore, the highly significant, negative temperature interaction at 5% (β = −0.0731, *p* < 0.001) proves that the RSBS polymer network actively restricts high-temperature fluidization, ensuring a more resilient and stable viscoelastic structure than the LSBS matrix under high thermal loads, especially while presenting properties of high SBS dosage.

It has been observed that the rutting parameter is not reliable in the case of modified binders [89,90] due to the excessively low strain applied during the test procedure and the linear viscoelastic nature of the measurements. It has been indicated that there is a need to incorporate nonlinear viscoelastic concepts and higher strain environment to properly simulate the behavior of modified binders, since rutting tests performed on asphalt mixtures did not correlate with the DSR parameters [20].

The Multiple Stress Creep and Recovery test (MSCR) was developed to account for higher strain levels and the nonlinear behavior of the material [91]. The test procedure is performed after the RTFOT ageing under two stress levels, including creep and recovery cycles, which provide a more representative response of the polymer-modified binder due to the delayed elastic effects of the rubbery modifier. The MSCR test introduces a new parameter describing the rutting resistance of the binder, namely J_nr_ (kPa^−1^), the non-recoverable creep compliance, determined under stress levels of 0.1 kPa and 3.2 kPa (calculated as the average value from 10 cycles at each stress level). AASHTO M332 [92] specifies threshold values of J_nr_ to classify different types of binders according to expected traffic loading, as follows:

- ≤4.5—Standard (4.0 according to MP19 [93]),
- ≤2.0—Heavy,
- ≤1.0—Very Heavy,
- ≤0.5—Extreme.

The J_nr_ parameter also allows for the calculation of the binder’s resistance to permanent deformation, expressed as J_nr,diff_ (%) and based on results obtained at both stress levels (2).

(2) $$J_{nr,diff}=\frac{J_{nr3.2}-J_{nr0.1}}{J_{nr0.1}}*100,$$

Research describing the correlation between asphalt mixture rutting performance (for example, ALF rutting) and binder rheology parameters has included both field and laboratory tests [94]. The correlation is typically expressed using a linear function and the coefficient of determination (R^2^), which shows more promising values for the DSR rutting parameter Jnr than DSR G*/sin(δ) in case of modified binders. This confirms the necessity of performing MSCR for a more accurate evaluation of modified binders [95,96,97,98].

The second parameter obtained from the MSCR is the percent recovery R, defined as the ratio of the recovered strain at the end of each cycle to the strain induced during creep. The results of R_3.2_ and J_nr3.2_ allow for the identification of the presence of a modifier in the bituminous binder.

**Figure 9:**
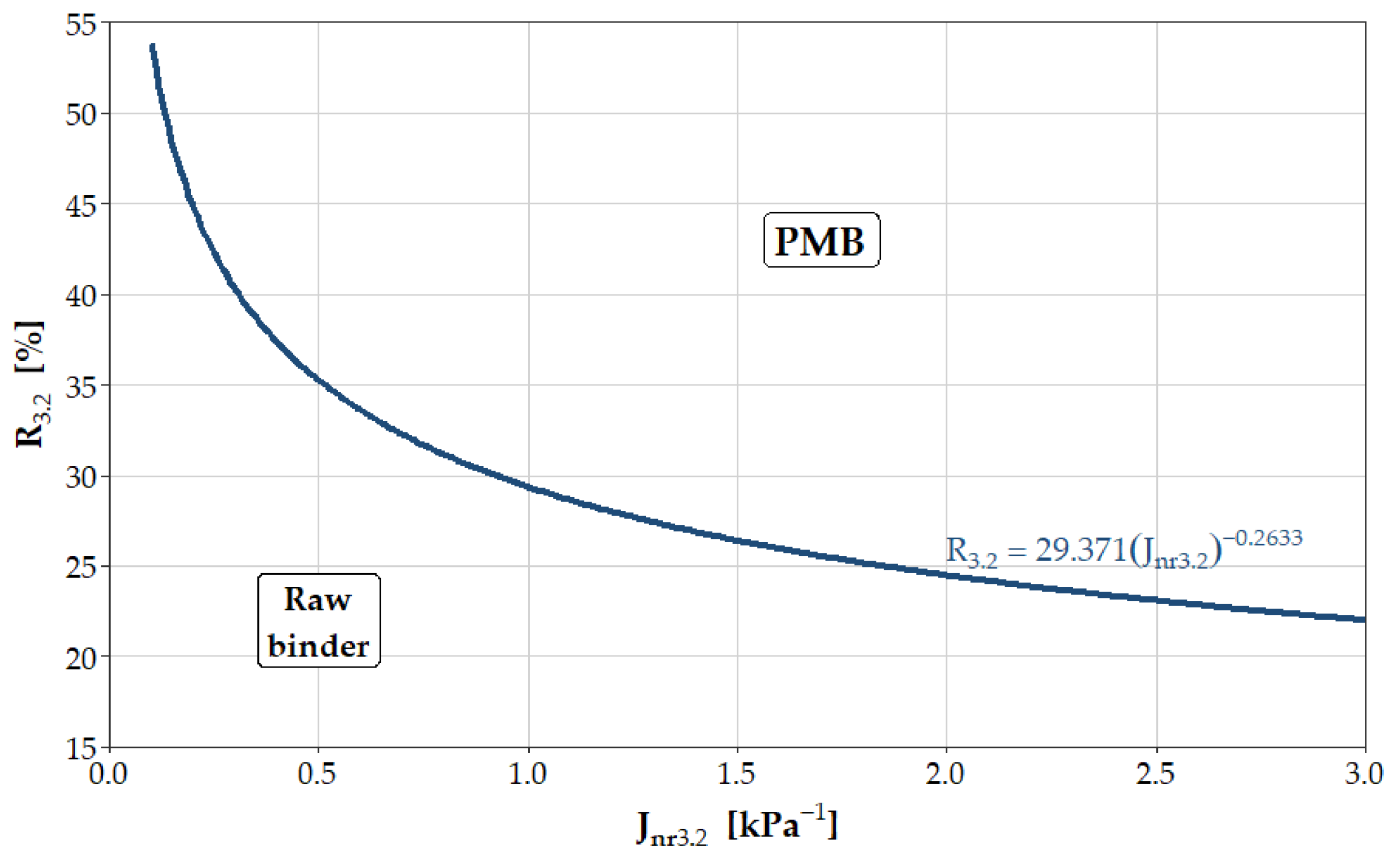
Curve R_3.2_(J_nr3.2_).

According to AASHTO protocols, the curve of R_3.2_(J_nr3.2_) establishes a boundary between modified binder (above the curve) and unmodified binder (below the curve), making it reliable method for determining the modification status of tested material (Figure 9).

Research confirms that SBS modified binders exhibit higher viscosity than raw binders, and the results of the rutting parameter typically allow PMB to be classified into very heavy or even extreme traffic conditions according to AASHTO [99,100,101], with improvements in percent recovery and compliance observed as the SBS content increases [102]. Nevertheless, it is crucial to evaluate the rheological properties of PMB and HiMA after field ageing to consider potential recycling strategies, which requires a comparison with current laboratory methods used to simulate binder ageing.

### 2.5. Ageing

The properties of PMB change over time due to oxidation, irradiation, heat and presence of other chemical compounds, and these processes become even more complex when the bitumen contains chemically active thermoplastic elastomer.

**Figure 10:**
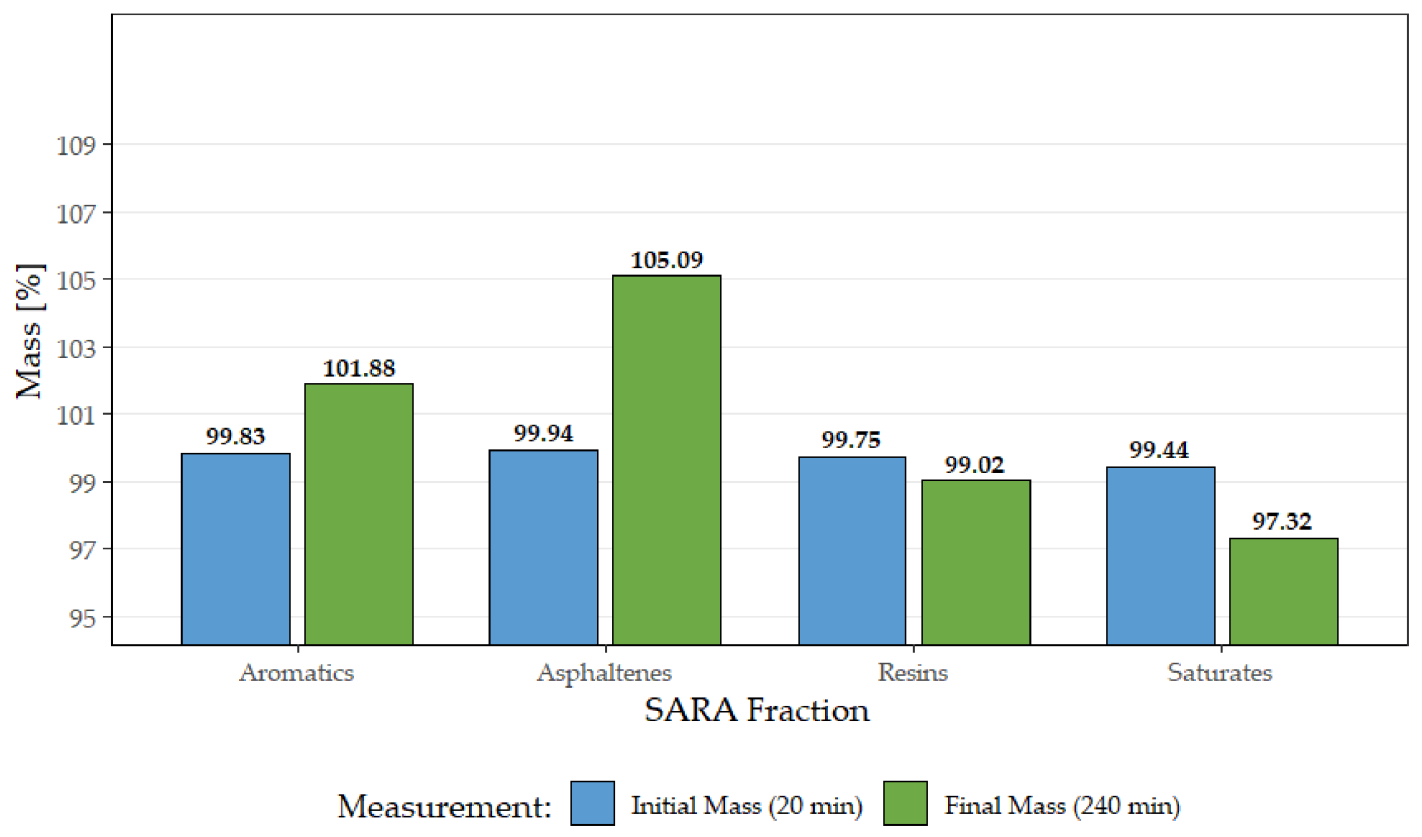
Graphical representation of mass changes in SARA fractions before and after RTFOT ageing conditions, based on [106].

Current methods for simulating technological and exploitation ageing of asphalt binders are, respectively. the Rolling Thin Film Oven Test (RTFOT) and the Pressure Ageing Vessel (PAV). Both methods are based on thermooxidation, with RTFOT conducted at 163 °C and PAV between 90 °C and 110 °C. These procedures allow the observation of changes in binder properties due to ageing under controlled laboratory conditions. The direct parameters of these tests are measured as mass changes before and after ageing. The mass can either increase or decrease during ageing process, depending on the composition of binder fractions (SARA). Binders with relatively higher content of light fractions tend to lose mass during ageing due to the evaporation of volatile components with weak bonds at elevated temperatures [103]. In contrast, binders containing more high-molecular-weight fractions often increase their mass during ageing process due to the oxidation of other compounds, such as benzene rings, and polycondensation in the aromatic fraction [104], or the formation of carbonyl and sulfoxide groups in asphaltenes, which leads to an increase in their content [105]. These changes in binder fractions are illustrated in Figure 10, which shows the results of thermogravimetric analysis of asphalt binder aged at 163 °C for 4 h [106]. The decrease in the resin fraction is caused by partial conversion to asphaltenes during oxidative condensation, while side chains break into low-molecular-weight volatiles [107]. Low-molecular-weight compounds are present in all binder fractions, but their loss is compensated by the formation of high-molecular-weight compounds; the interplay of these two phenomena results in either an increase or a decrease of the total mass after ageing [108].

The procedure separating each binder fraction was performed according to ASTM D4124 [109]. Process involves precipitating asphaltenes with an alkane solvent like n-heptane or iso-octane to dissolve the soluble petrolenes in warm conditions, filtration of the solution and separating the asphaltenes to dry and weigh. Next, a glass chromatographic column is packed with dry alumina (such as calcinated F-20 or CG-20 alumina), then wet and equilibrated using n-heptane. The remaining petrolenes are dissolved in n-heptane and introduced to the top of prepared alumina column. N-heptane is used to elute the column and collects the saturates; then, this step is repeated by subsequent polar solvents like toluene to obtain aromatics and resins. The influence of SBS in this study was calculated using ageing reaction kinetics analysis.

**Figure 11:**
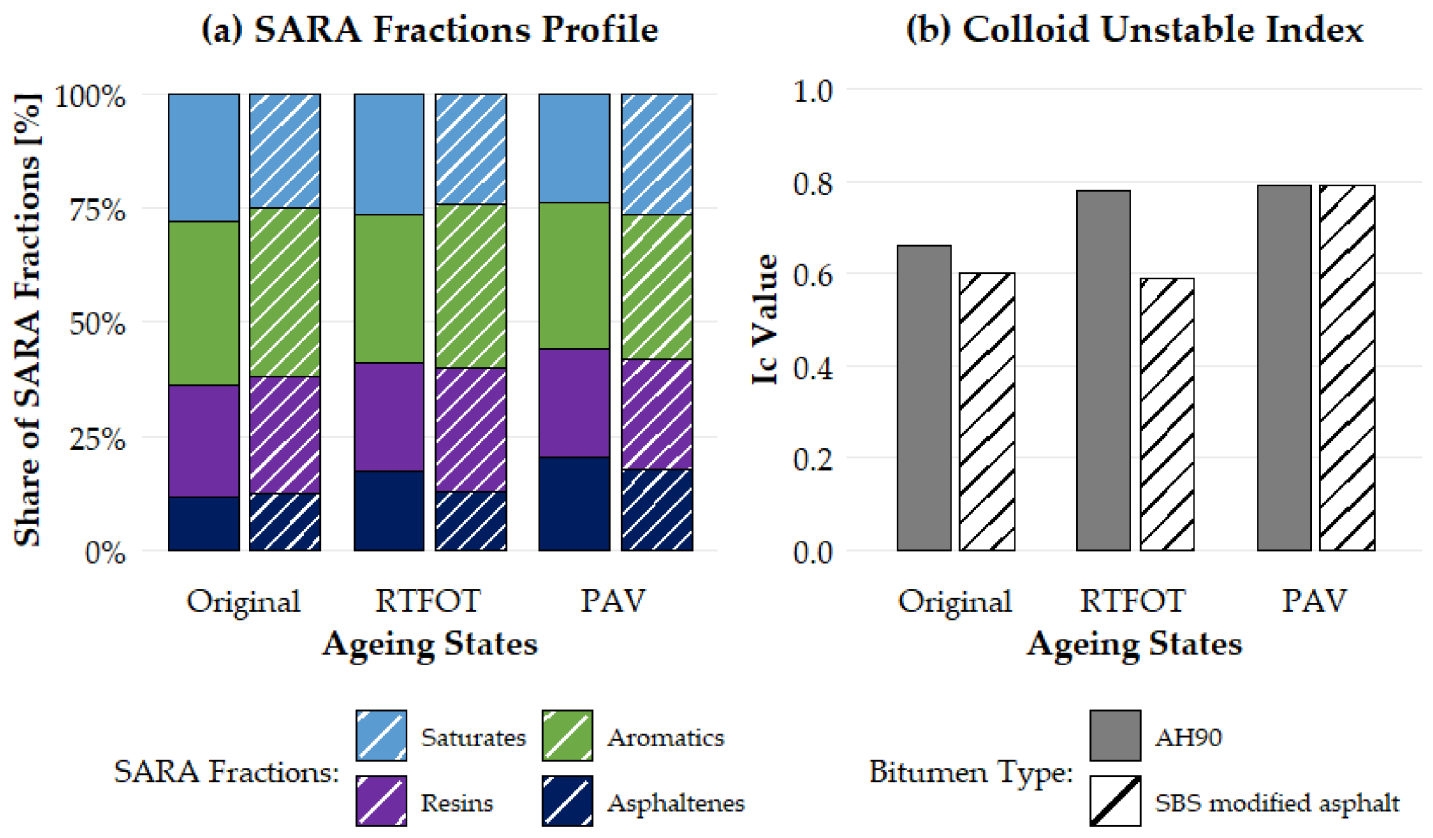
SARA analysis for raw and SBS-modified binder before and after the ageing process, based on [108].

Figure 11 summarizes the fractional analysis results for unmodified binder and binder modified with 3% SBS in the RTFOT and PAV ageing tests [108]. After short-term ageing, the amounts of saturated and aromatic compounds decrease for both binders, while the amount of asphaltenes increases. This behavior is typical for asphalt binders. However, after long-term ageing, differences in the behavior of the two binders are visible. For the unmodified binder, the saturated fraction content continues to decrease, whereas for the modified binder, it significantly increases. This is due to the degradation of the dispersed SBS polymer, which had previously absorbed the light fractions of the binder and no longer serves as a modifier. The aromatic fraction content gradually decreases for both binders, while the asphaltene fraction content increases after both ageing methods. The resin content is inversely proportional to the saturated fraction content and decreases after the field-ageing simulation.

**Figure 12:**
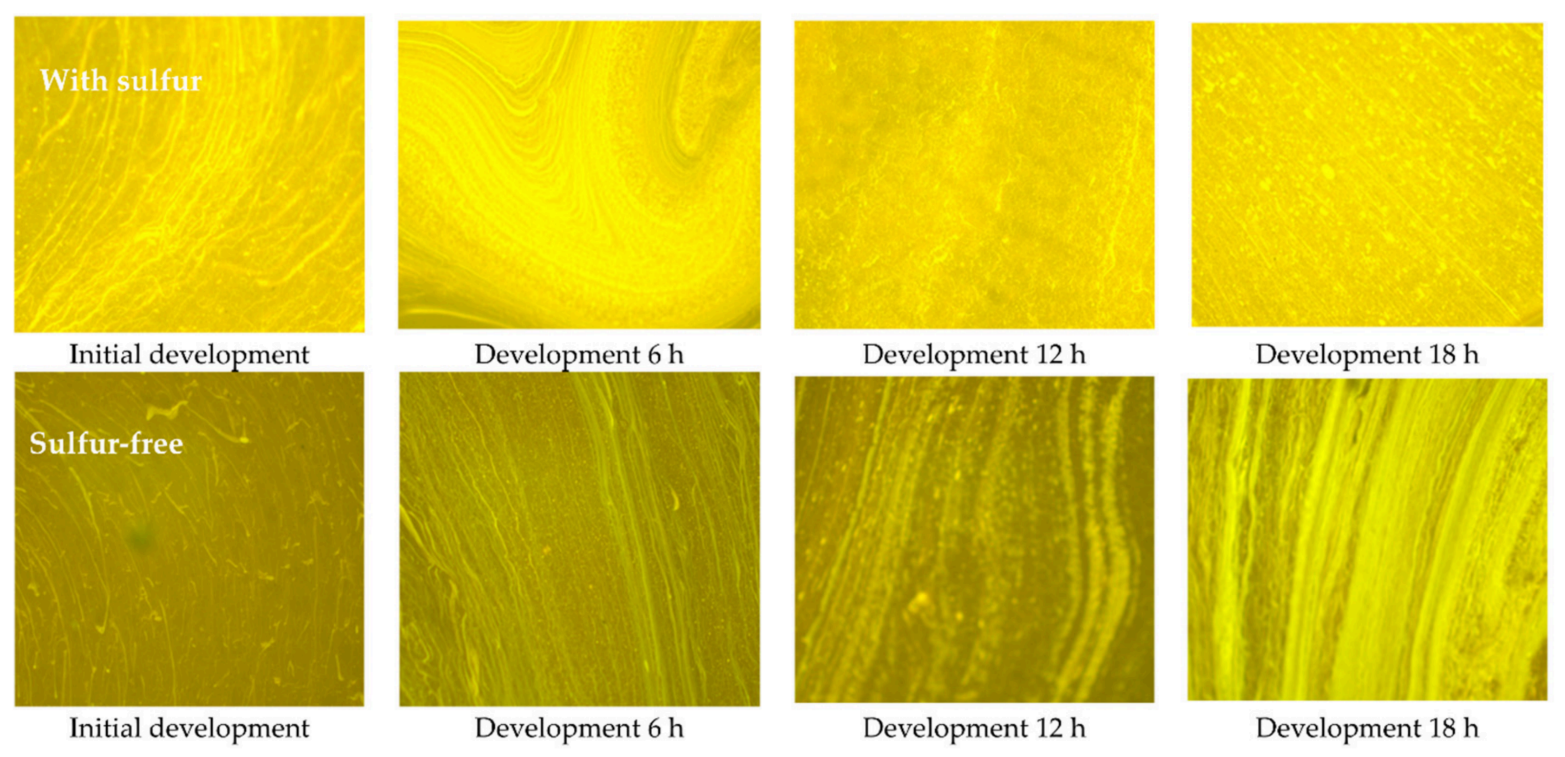
Fluorescence microscopy tests of SBS-modified bitumen with and without sulfur added during high-temperature development, reprinted from [110] under the terms of open access (CC-BY 4.0 license).

The properties of aged binder can be studied using previously described parameters (penetration, softening point, Fraass breaking point), as well as spectroscopy methods, microscopy and rheological measurements. PMB ageing causes degradation of the polymer [110,111,112,113], the loss of elastic properties and enhanced interactions with binder compounds [114,115]. It also increases the viscosity of the bitumen [116]. Oxidation of the binder leads to the formation of new sulfoxide and carbonyl groups, which can be monitored by spectroscopy and used to quantify the degree of ageing [117]. It was observed that short-term ageing of the binder has a stronger influence on the formation of sulfoxide groups than carbonyl groups [118]. Microscopy observations of SBS polymer during ageing yield inconsistent results, as the changes depend on several complex factors, including temperature, duration, irradiation and the presence of other reactive agents, such as sulfur or reactive oxygen species. One of the indicators of SBS polymer structure degradation is the infrared index IB/S, calculated as the ratio of the polybutadiene peak intensity to the polystyrene peak intensity measured during spectroscopy, since ageing primarily affects the polybutadiene structure [119]. The presence of other compounds can alter the polymer’s behavior during ageing. For example, the addition of sulfur as a compatibilizing agent promotes cross-linking of the SBS polymer (Figure 12) [110]. Additional sulfur results in a more visible cross-linked polymer network during the initial development of the binder and after 6 h under high temperature and shear. However, the polymer matrix with added sulfur degrades after longer periods, likely as a result of C-S bonds created during sulfurization. In contrast, PMB without sulfur exhibits the progression of cross-linking under high-temperature and shear conditions, enhancing its viscoelastic properties.

**Figure 13:**
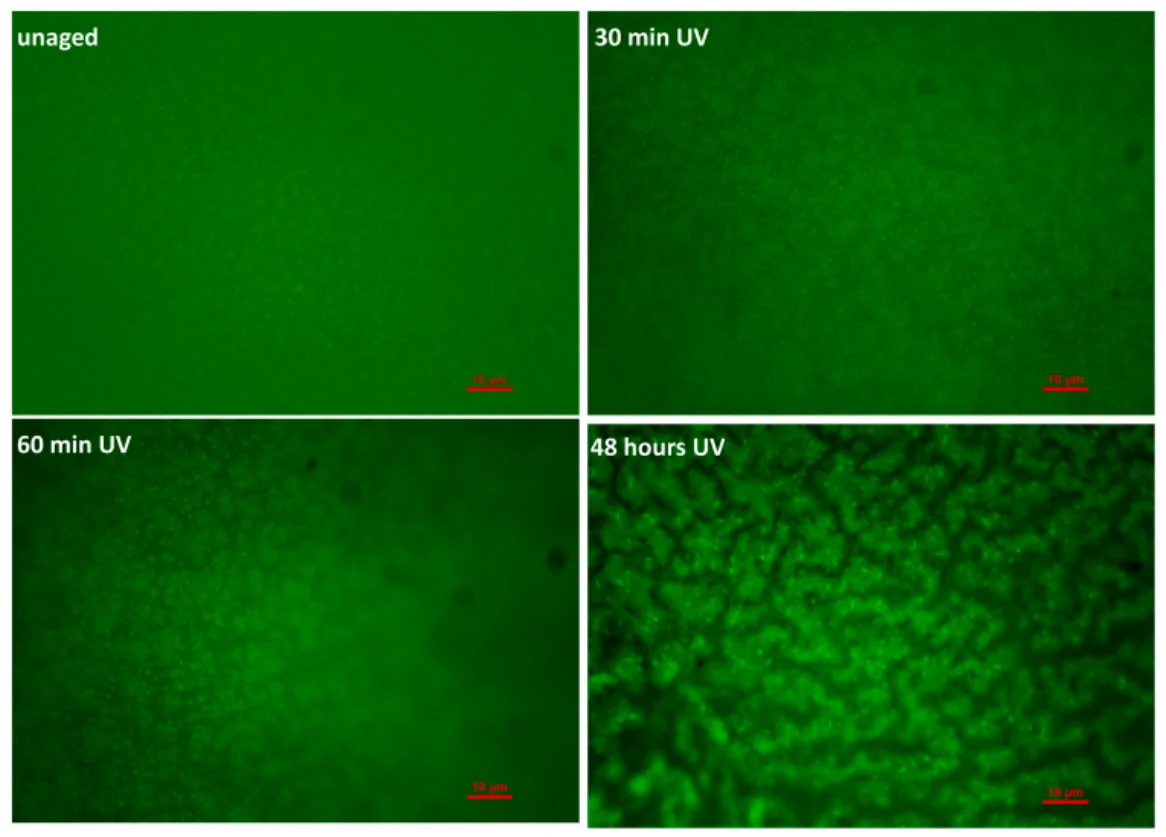
Fluorescence microscopy images of bitumen surface affected by UV irradiation, reprinted from [84] under the terms of open access (CC-BY 4.0 license).

In addition to thermooxidation, field ageing of asphalt binders may be caused by UV radiation, which is not included in the long-term laboratory PAV process. This limitation implies that laboratory simulations do not fully reproduce real field ageing conditions. UV radiation leads to the photochemical degradation of asphalt mixtures [120,121]. It has been shown that the influence of UV radiation and Reactive Oxygen Species (ROS) occurring on the pavement surface influence the chemical composition of the binder during ageing differently than RTFOT and PAV methods [84]. Although FTIR tests indicate that under UV radiation SBS cross-linked structure degrades [122,123], as evidenced by the decrease of polybutadiene C=C bonds and the increase of functional groups containing oxygen, Figure 13 [84] shows a visible cross-linked structure formed after exposing the SBS modified binder to UV radiation—an observation not previously reported by the research. However, the direct influence of each ageing factor as separate has remained unresolved, since the separation of high temperature, UV radiation and oxidation has not yet been done.

The inconsistency highlights the complexity of the reviewed material and its susceptibility to the changes in the environment, causing challenges with potential recycling.

## 3. Ageing of Hot Asphalt Mixture with PMB and Recycling

Asphalt mixture ageing is primarily driven by the ageing of the bitumen, however, aggregates also influence the process due to their chemical composition and adhesive properties [124]. The SHRP A-383 procedure was developed for asphalt mixtures, corresponding to the following ageing methods for asphalt binders (RTFOT and PAV) [125]:

- STOA—Short-Term Oven Ageing (technological)—4 h at 135 °C,
- LTOA—Long-Term Oven Ageing (exploitation)—120 h at 85 °C.

Numerous publications utilize the above-mentioned methods, including their modified versions. In 2016, a study was published comparing the ageing times for the STOA and LTOA methods, as well as the ageing temperature (LTOA method only). To compare the behavior of all mixtures after ageing, the dynamic modulus was measured using the AMPT apparatus, along with the phase shift angle [126]. The study showed that extending or shortening the short-term ageing time does not significantly affect the dynamic modulus of the asphalt mixture. This suggests that during STOA, the primary ageing factor is the “shock” experienced by the material when exposed to high temperatures. In contrast, for LTOA, both increasing the ageing time and raising the temperature visibly affect the properties of the asphalt mixture. Higher temperatures and longer service ageing times result in an increase in the dynamic modulus and, consequently, greater mixture stiffness, indicating more pronounced ageing effects. However, the authors emphasize that the ageing factors used in these methods do not adequately account for atmospheric influences, including UV radiation, similarly to bituminous binder ageing methods.

For mixtures containing modified binders it is expected that larger quantities of RAP could be used to produce new mixtures. This is due to the similar properties of high stiffness bituminous binders and binders aged in the existing pavements, which allows for more advantageous unification of materials [127].

**Figure 14:**
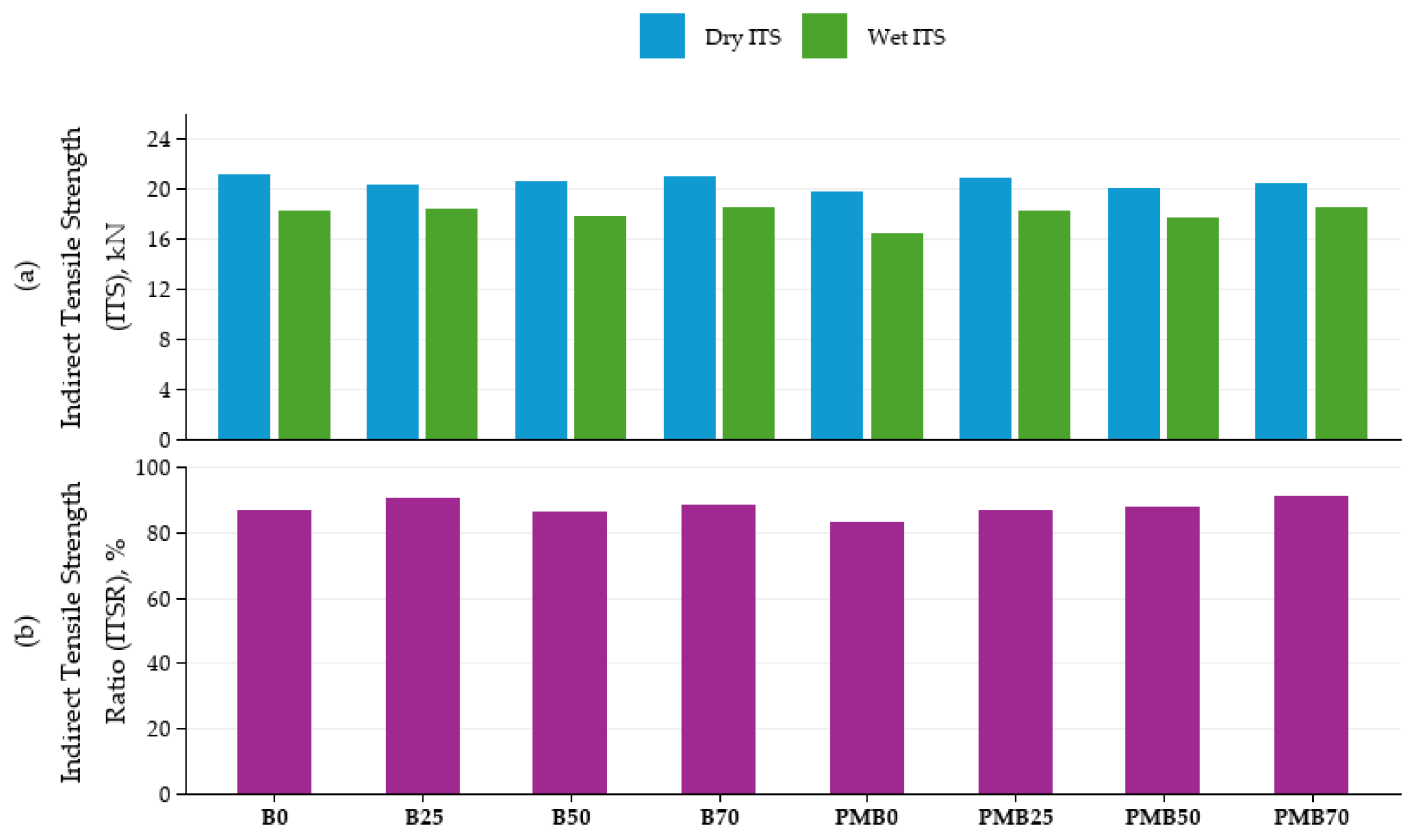
ITS results for dry and wet sample sets (**a**) and ITSR (**b**) based on [127].

**Figure 15:**
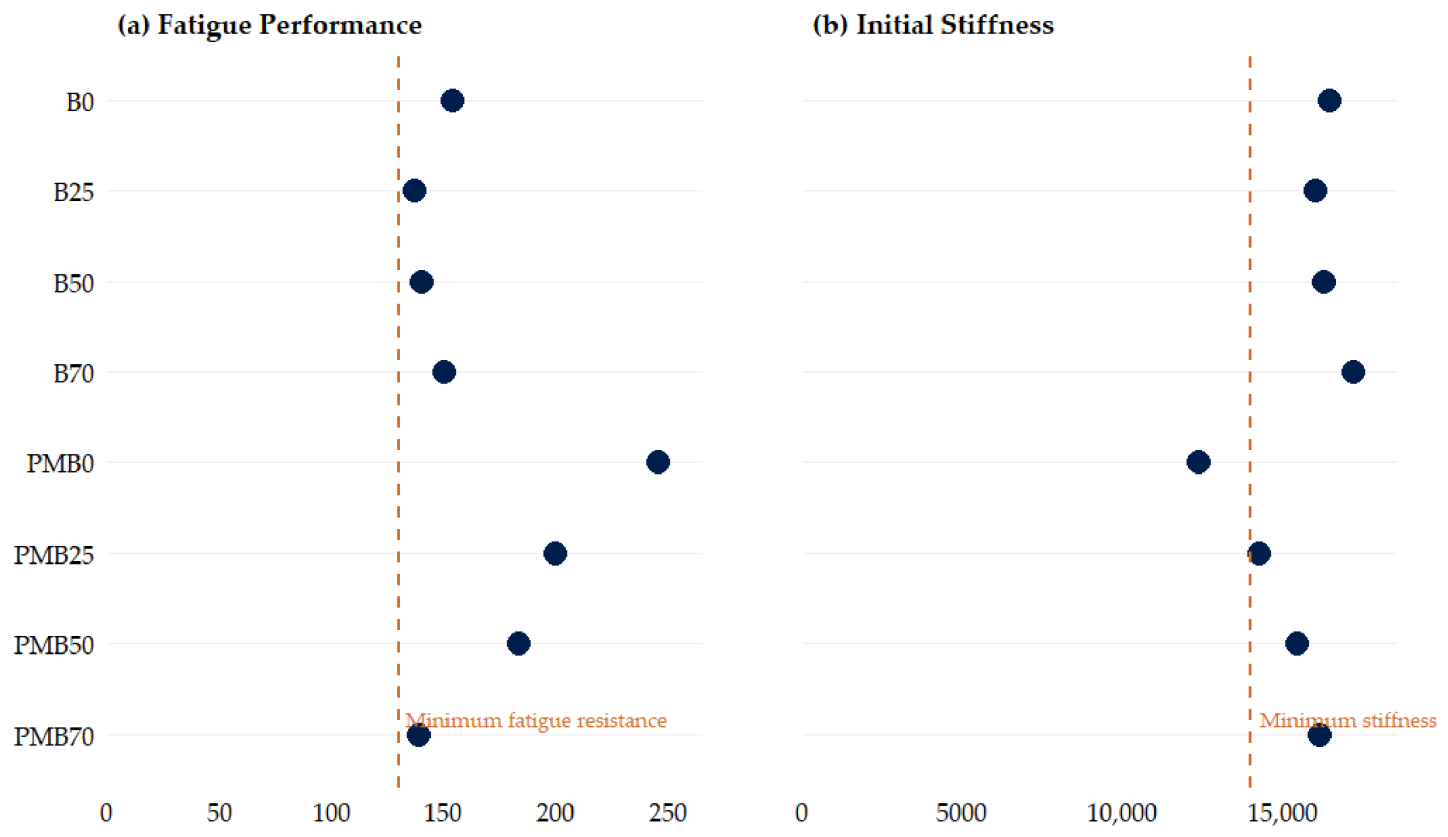
Results of initial stiffness at 100th cycle (**a**) and fatigue performance after 1,000,000 cycles (**b**) based on [127].

Izaks et al. [127] conducted tests using RAP and two asphalt binders: hard bitumen 20/30 and highly SBS-modified 25/55-80 bitumen. For each mixture, 0%, 25% and 50% of RAP were added. The performance parameters assessed to evaluate the suitability of reclaimed asphalt included ITS and ITSR tests (Figure 14), fatigue and stiffness (Figure 15). All mixtures demonstrated high resistance to water (above 80%). Mixtures containing HiMA exhibited lower stiffness than mixtures with hard bitumen, and the mixture without RAP failed to meet the required stiffness level. Despite this, all mixtures from the HiMA series met the fatigue life requirements, including the mixture containing 70% RAP, which also met the stiffness requirement. It should be noted that these results were obtained without the addition of rejuvenator or soft bitumen, which are known to positively influence properties such as water resistance and fatigue performance.

To investigate the relationship between the mechanical parameters at different stages of the asphalt mixtures’ lifecycle, a Pearson correlation analysis was performed between the initial stiffness (at the 100th cycle) and fatigue strain (at 1,000,000 cycles). The statistical significance of the correlation coefficients (r) was evaluated using a two-tailed *t*-test with a significance level of α = 0.05. The statistical testing revealed fundamentally contrasting structural behaviors between the conventional and polymer-modified asphalt mixtures:

- Conventional asphalt mixtures (bitumen B): A moderate positive correlation coefficient was obtained (r = 0.631). However, the correlation did not reach the threshold of formal statistical significance (*p* = 0.3691, *p* > 0.05). This lack of formal significance is primarily attributed to the small sample size (n = 4) inherent to extensive, long-term fatigue testing. Nevertheless, the result indicates a noticeable technological trend where a stiffer initial aggregate–bitumen mixture tends to accommodate higher fatigue strains before failure under standard conditions.
- Polymer-modified asphalt mixtures (bitumen PMB): An exceptionally strong negative correlation was observed (r = −0.969). Despite the limited sample size (n = 4), this correlation was found to be statistically significant (*p* = 0.0315, *p* < 0.05). This statistical evidence confirms a strict, inversely proportional relationship between the two parameters. From an engineering standpoint, it visibly demonstrates that an excessive increase in the initial stiffness of polymer-modified mixtures severely compromises their long-term flexibility. While the polymer network enhances performance, a matrix that is too rigid from the start becomes brittle under prolonged cyclic loading, resulting in a dramatic reduction in fatigue strain resistance at 1,000,000 cycles.

During the tests, the physical and rheological parameters of the high modulus asphalt concrete (HMAC) were assessed, and in batches 3 and 4, the limiting value of recycled asphalt addition was determined, allowing for the production of the mix without the addition of rejuvenators. The uniformity of reclaimed asphalt distribution in the HMAC was also verified. In this study [126], however, RAP did not contain aged SBS-modified bitumen; instead, PMB was used as virgin binder. Therefore, no information was provided regarding the recycling of aged PMB itself. In recent years, there has been very little research containing mixtures with PMB aged in real-life conditions, since these materials have high fatigue resistance and are milled far less than mixtures with unmodified binders.

**Figure 16:**
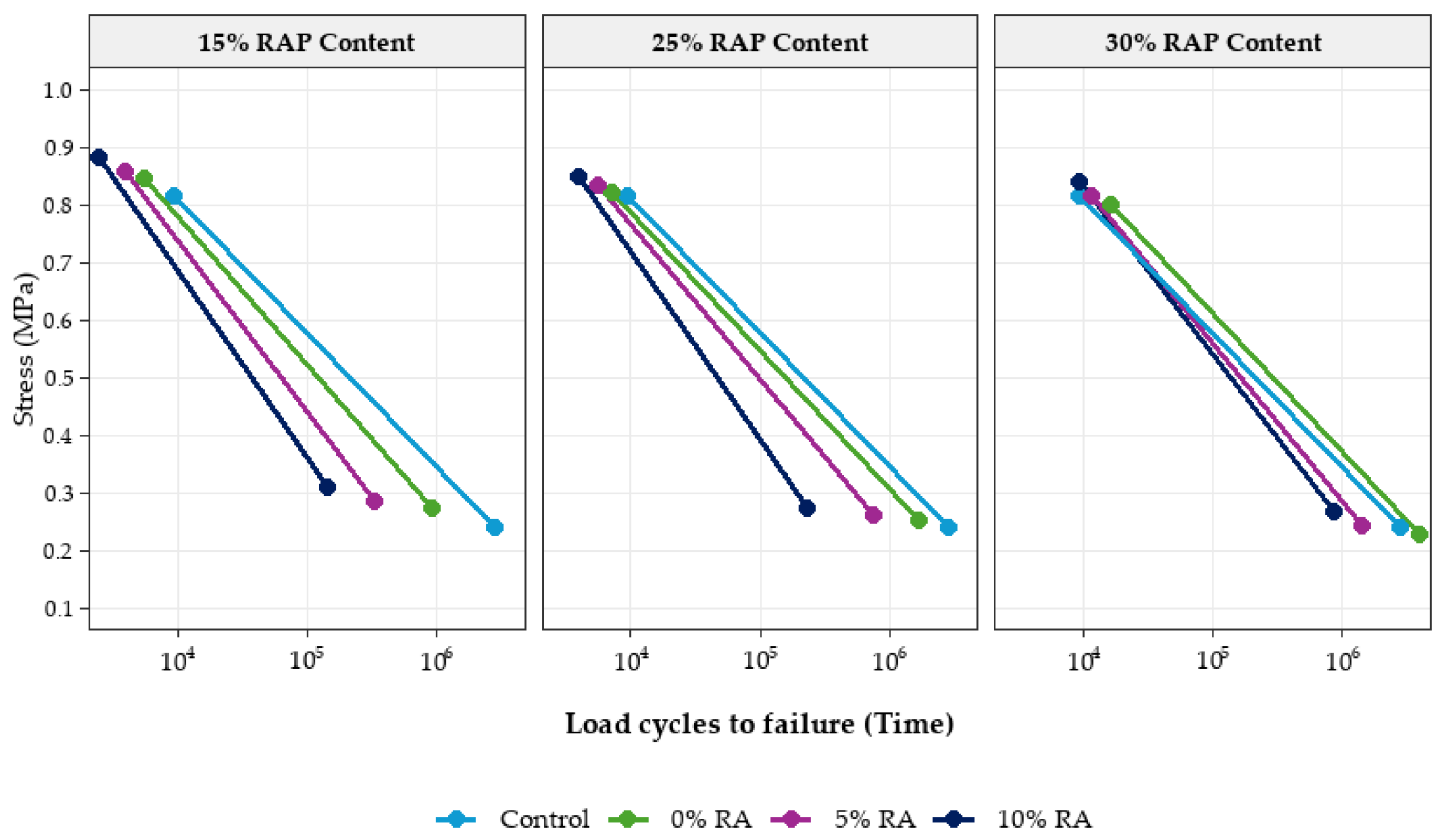
Fatigue for mixtures containing different additions of RAP and rejuvenator based on [128].

One of the few instances of research incorporating RAP from an asphalt mixture containing an SBS polymer-modified binder is shown in Figure 16 [128]. The results indicate that the presence of RAP decreases the fatigue performance of the asphalt mixture, whereas the addition of a rejuvenator improves its properties. These findings are consistent with those reported for mixtures containing unmodified asphalt binders.

To further advance the engineering interpretation, a Pearson correlation analysis was conducted directly on the slope parameters (17a) derived from the fatigue regression equations (σ = a × log(N) + b). The statistical test revealed an exceptionally strong and highly significant negative correlation between the rejuvenator dosage (0%, 5% and 10% RA) and the fatigue line slopes (r = −0.905, *p* = 0.0008, *p* < 0.001). This statistical evidence confirms that the chemical interaction of the RA additive fundamentally and predictably alters the stress-sensitivity profiles across all investigated RAP content levels.

From a mechanical perspective, the systematic shift toward more pronounced negative slope values indicates that while the rejuvenator increases the material’s sensitivity to stress changes over prolonged cyclic loading, this behavior is concurrently counterbalanced by a significant upward shift in the intercept boundary (b). The statistical validation proves that the rejuvenation process successfully restores the viscoelastic properties of the highly aged aggregate-bitumen mixture. By transitioning the brittle RAP mortar into a more compliant, ductile material, the additive effectively delays the coalescence of micro-cracks under prolonged fatigue cycles.

**Figure 17:**
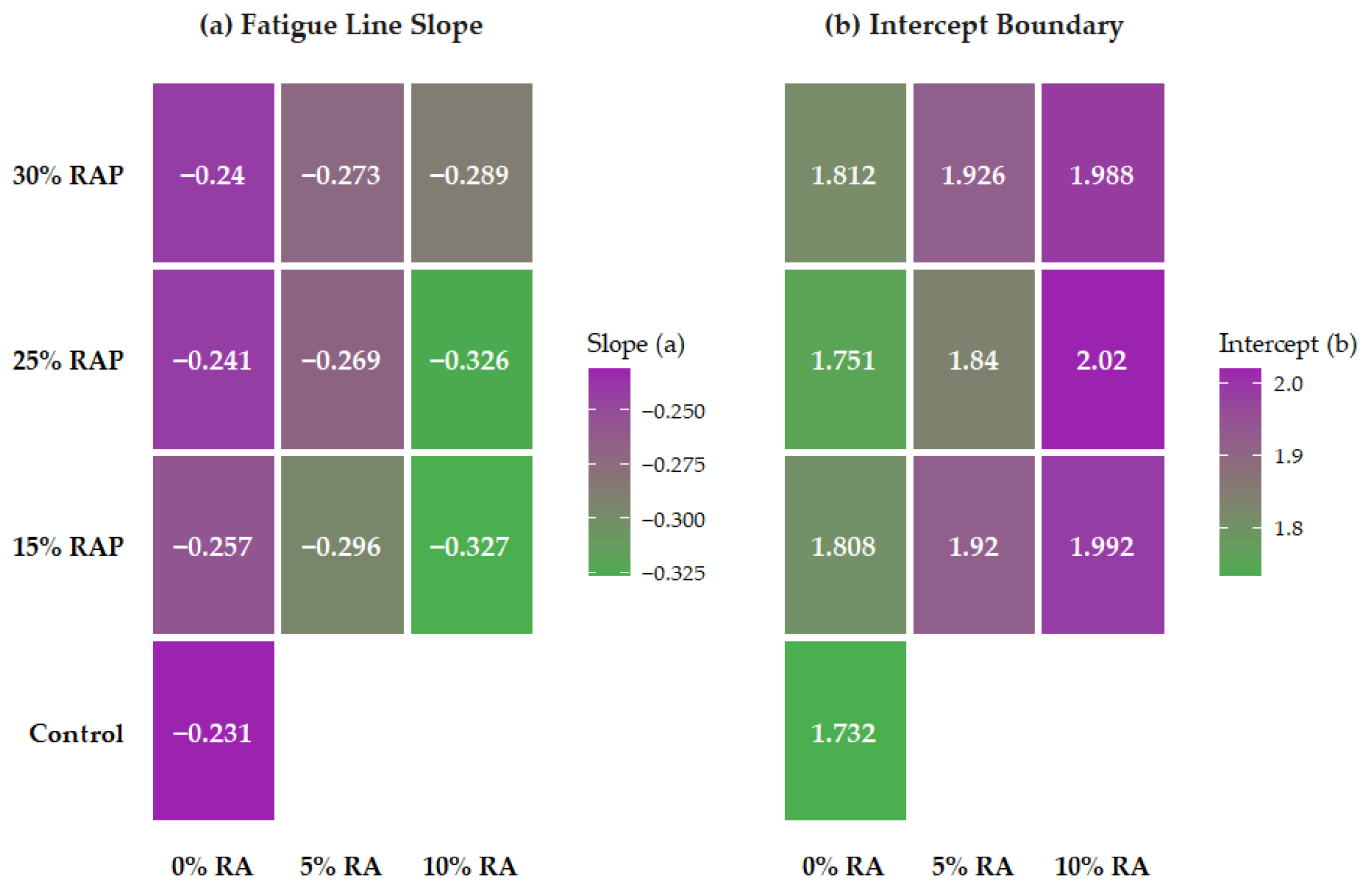
Slope (**a**) and interception (**b**) heatmap based on data from [128].

To properly compare the parameters of the regression model, Figure 17 presents slope and interception heatmap between the mixtures. The inclusion of the Control mixture as a baseline reference row at the bottom of the parametric matrix reveals several critical insights regarding the mechanics of binder rejuvenation.

First, a phenomenon of extensive rejuvenation influence is observed on the intercept boundary panel (Figure 17b). The unmodified Control mixture displays the lowest initial intercept value (b = 1.732). Upon the introduction of 10% RA, the intercept values for all RAP-inclusive matrices experience a dramatic growth, approaching or exceeding a threshold of 1.99. This indicates that the chemical interaction between the aged polymer-modified mixture, the residual SBS network, and the rejuvenator agent yields an upgraded skeletal cohesion that surpasses the fatigue resistance of the pure control material, even though it presents lower slope parameter, what indicates higher stress sensitivity. The heatmap successfully identifies a technological optimum at the 25% RAP threshold under 10% RA dosage., where the intercept value reaches absolute highest value (b = 2.020).

The slope heatmap (Figure 17a) unveils a stabilizing effect of high recycled content configurations. At the maximum 10% RA dosage, the 30% RAP mixture maintains a less pronounced negative slope (a = −0.289) compared to its 15% RAP counterpart (a = −0.327). This demonstrates that at 30% of RAP content, the highly rigid, locked structure of the recycled aggregate framework acts as a mechanical constraint. It mitigates the excessive compliance induced by the high dosage of the rejuvenator, thereby stabilizing the mixture’s overall stress sensitivity.

**Figure 18:**
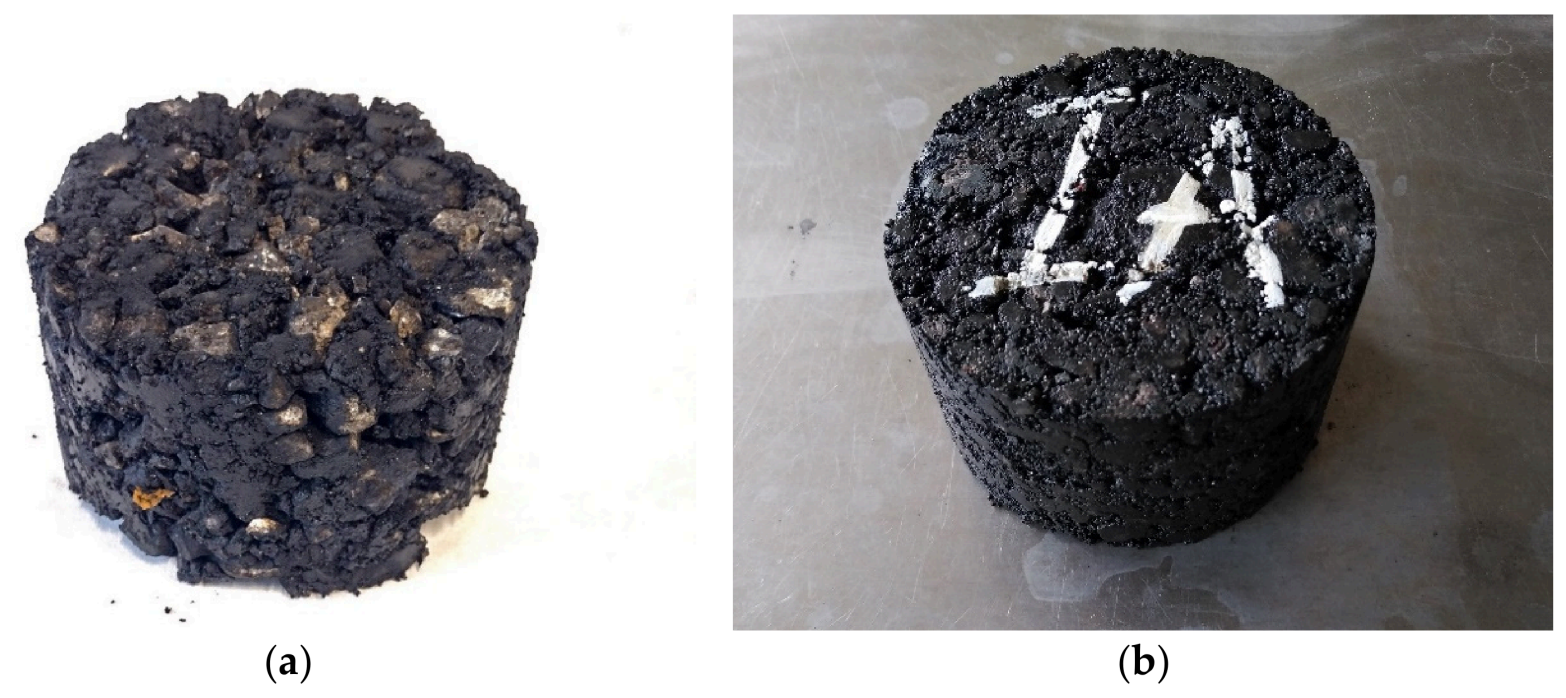
Marshall compacted sample of the asphalt mixture containing PMB 45/80-80 (**a**) after ageing at 200 °C compared with the (**b**) sample without additional conditioning [131].

Considering polymer chain reactions, the phenomenon of excessive cross-linking should be highlighted. According to previous studies, it was explained due to an overdose of a cross-linking agent or when the asphalt binder or mixture is exposed to excessively high temperatures [129]. This can lead to bitumen gelation, resulting in disturbances to the colloidal system and reduced binder stability [130]. The issue of overheating is particularly critical when RAP containing PMB is used in the production of new pavements. During processing, reclaimed asphalt may locally reach temperatures above 200 °C, which can significantly affect the properties of the final asphalt mixture (Figure 18) [131].

This problem is relevant at asphalt mixture plants, where RAP is incorporated to the mixture using two main technologies [132]:

- cold dosing—cold RAP added to aggregate mixture, heated at higher temperature than the target production temperature,
- hot dosing—RAP is preheated prior to its incorporation to the mixture using specifically designed equipment.

**Figure 19:**
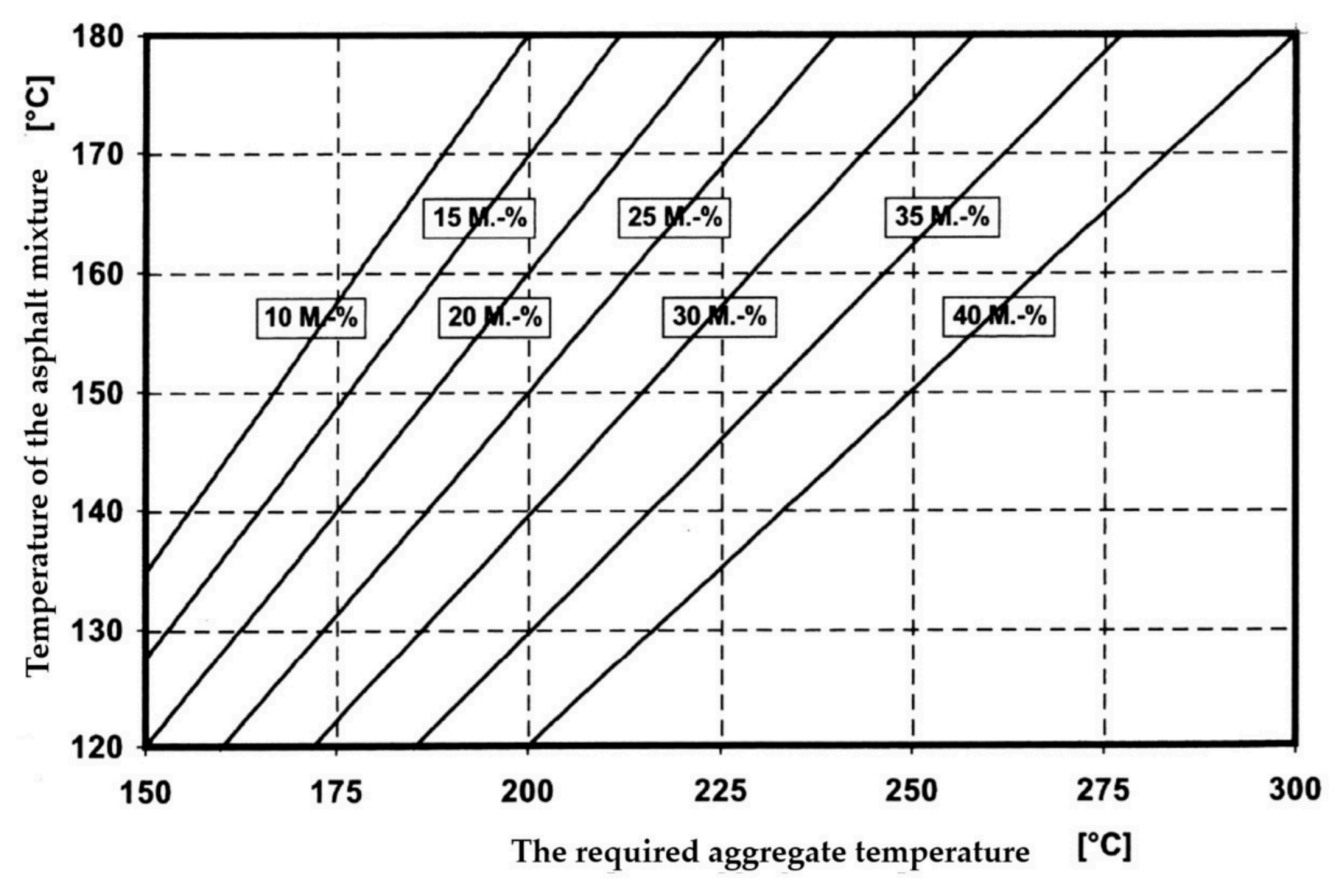
The required aggregate temperature in Poland depending on the percentage of cold and dry asphalt granulate in the asphalt mixture (horizontal axis—required temperature of the aggregate, vertical axis—temperature of the asphalt mixture) [134,136].

According to EAPA [4], as of 2023, some mixing plants still lacked the proper equipment required for hot dosing RAP. In Poland, the majority of asphalt mixing plants do not have proper technology. The cold dosing method does not guarantee full homogenization of the RAP and the mixture due to significant temperature differences between the components [133]. Moreover, there is a risk of overheating the RAP binder, as aggregate temperatures may reach up to 300 °C (Figure 19) [134]. In case of PMB, overheating can severely damage the polymer matrix, causing degradation and/or excessive cross-linking, being the main cause of problems with compacting the asphalt mixture. It is important to emphasize that, in addition to cohesive failure associated with binder ageing, adhesive failure may also occur. Adhesive failure refers to the interaction between the binder and the aggregate. Although aggregates themselves do not undergo ageing, their chemical composition and porosity significantly influence the ageing behavior of the mixture. In most cases, adhesion-related issues manifest as stripping. However, overheated mixtures containing HiMA may exhibit the opposite problem. Therefore, it is necessary to determine which of the described phenomena causes each problem with HiMA RAP structure and compaction–polymer degradation, uncontrolled cross-linking or adhesive failure [135].

Regardless of the method used to recycle RAP, in most cases additional materials should be used to restore the original properties of the asphalt binder prior to ageing. These materials may include [137]:

- soft bitumen,
- rejuvenating agents.

Rejuvenators are agents that restore the lighter fractions of bitumen, such as saturates and aromatic oils. The rejuvenator content should be carefully calculated, as there is a risk of disrupting the initial binder properties and impairing certain characteristics, such as the mixture’s rutting resistance. The addition of a rejuvenator should improve fatigue performance, low-temperature properties and resistance to low-temperature cracking while maintaining high-temperature resistance [137,138].

**Figure 20:**
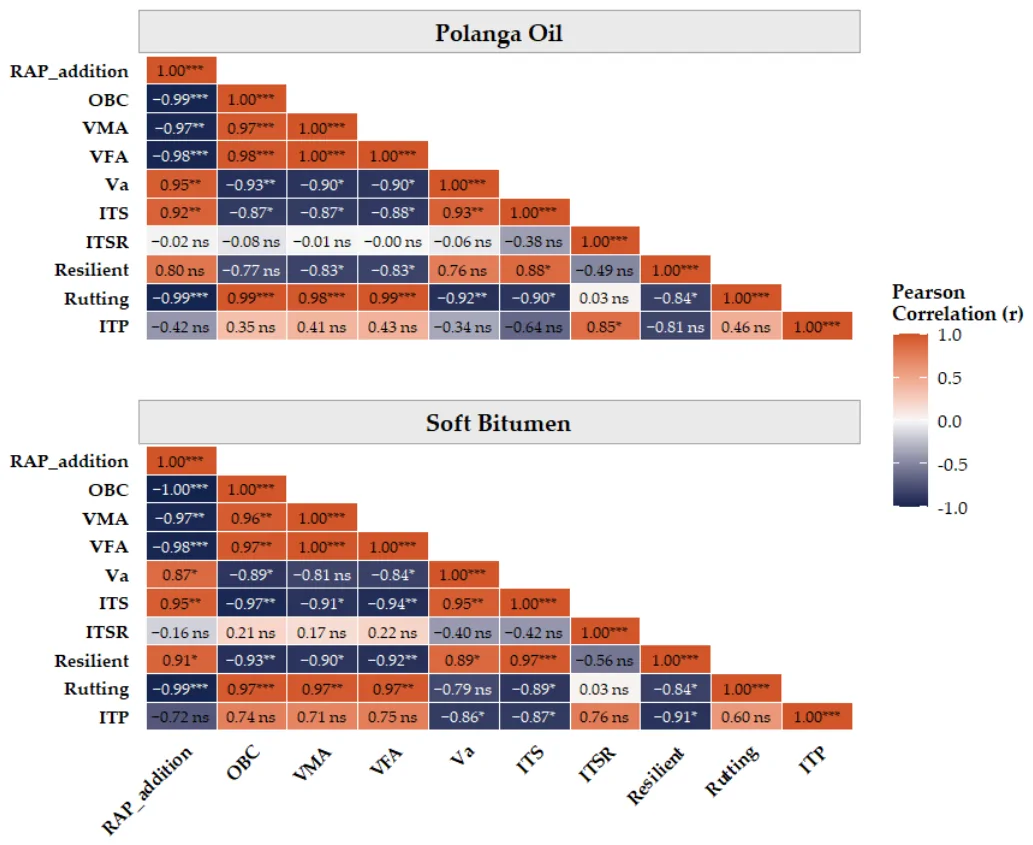
Correlation heatmap (Pearson analysis, ***—*p* < 0.001, **—*p* < 0.01, *—*p* < 0.05, ns—non-significant) based on data from [139].

The comparison of soft bitumen and rejuvenating agent is crucial to determine justification for use complex chemical compounds instead of already broadly produced soft bitumen. Pradhan, S.K.; Sahoo, U.C. [139] conducted a study where new asphalt mixture was produced using different contents of RAP. For each RAP content (30%, 40%, 50%, 60% and 70%), rejuvenation additive was added by mass of aged binder present in RAP—soft bitumen or polanga oil. The correlation heatmap (Figure 20) and ANCOVA model comparison (Table 5) were created based on original data to expand the author’s ANOVA analysis. This statistical approach allows for a direct, rigorous verification of the previous conclusions by isolating compounding variables and quantifying structural trends.

The original study concluded that the optimum binder content (OBC) decreased with the inclusion of the rejuvenator, leading to a saving of fresh virgin bitumen. While standard ANOVA treated this purely as an isolated volumetric phenomenon, the new vertical correlation heatmaps explain the inner structural mechanisms. For the soft bitumen rejuvenation, OBS shows a perfect inverse relationship with RAP addition (r = −1.00 ***), proving that the softer bitumen acts strictly as geometric spacer displaced by the aged RAP fraction. Conversely, within the polanga oil matrix, a distinct chemical equilibrium is captured (r = −0.99 ***). Here, the rejuvenating agent actively diffuses into and restores the highly clustered asphaltenes of the old RAP bitumen, lowering the overall global demand for fresh virgin bitumen. This microstructural behavior provides clear statistical validation for the documented binder savings. Furthermore, the invariant behavior of air voids (Va ≈ 4.0%) across both technological panels validates that the volumetric skeleton remained highly consistent (r = 1.00 *** for polanga oil and r = 1.00 *** for soft bitumen).

The primary advantage of polanga oil over the soft bitumen strategy is revealed when comparing their baseline shifts against the control formulation. The soft bitumen approach reduces resilient modulus (β = −693.322, ns) but triggers an explicit reduction in rutting resistance (β = −0.569, *p* < 0.05). In contrast, polanga oil cuts the global stiffness trajectory twice as effectively (β = −1434.150, ns) while maintaining excellent stability against permanent deformation (Rutting = +0.094, ns). While the original authors concluded that the rutting potential of mixtures rejuvenated with polanga oil is higher than that of soft bitumen mixtures, the ANCOVA model shows this is a minor, non-significant change. Crucially, all formulations satisfy the regulatory safety limit of 12.5 mm after 20,000 wheel passes.

The mechanical consequences of RAP addition are mapped by the RAP Content Linear effect. As RAP content increases, the mixture experiences clear structural hardening. This is proven by significant linear increases in indirect tensile strength (ITS = +1.453, *p* < 0.001) and resilient modulus (Resilient = +3065.827, *p* < 0.05), driving the dynamics stiffness toward its peak near 8000 MPa at high RAP contents. For the ITS parameter, the quadratic effect shows a significant negative value (−0.382, *p* < 0.05), proving that the strength curve safely flattens out at extreme RAP contents. This general hardening reduces moisture resistance (ITSR = −0.189, *p* < 0.001) and intermediate temperature cracking performance (ITP = −0.008, *p* < 0.001). Despite this decay, the original authors noted that all mixtures achieved the minimum required ITSR, showing no active moisture damage threats.

The advanced model explains this behavior as a clear, controlled viscoelastic compromise. While soft bitumen shifts the intermediate temperature cracking performance (ITP) baseline by a modest β = +0.221 (*p* < 0.01), polanga oil exactly doubles this elastic recovery effect, yielding a highly conservative advantage of β = +0.441 (*p* < 0.001). The slight increase in rutting potential found in the polanga oil mixtures (β = 0.0094, ns) is a direct, statistically verified parameter of cracking resistance. Most importantly, the “RAP content × Rejuvenator interaction” reveals a statistically significant protection vector for polanga oil within the ITP parameter (+0.003 *), whereas the main effects remain parallel. This confirms that polanga oil actively delivers a reliable, structurally stable viscoelastic structure at intermediate temperatures, actively preventing from catastrophic pavement cracking at high RAP contents without violating regulatory permanent deformation safety boundaries.

In summary, while both recycling strategies maintain identical volumetric stability with constant air voids, polanga oil allows for higher fresh bitumen savings by chemically active reduction of the OBC. Furthermore, while soft bitumen provides lower rutting depths, polanga oil delivers twice the efficiency in reducing resilient modulus and enhancing intermediate temperature cracking resistance.

Since most rejuvenating agents target bitumen fractions, they do not precisely restore the structure of the polymer matrix in PMB [140,141]. However, it does not mean that they have no positive impact on aged PMB. Rejuvenating agents have been proven effective in improving the properties of recovered SBS-modified binders, as they restore the balance of the binder similarly to unmodified binders [142].

In addition to restoring optimal proportion of bitumen fractions, PMB rejuvenators must also address polymer chain reactions [143]. Authors differ in their opinions regarding the cause of the PMB undesirable behavior. Some claim that elevated temperatures promote deeper cross-linking reactions [129], especially over 200 °C, where the process becomes uncontrollable [144]. Others describe the degradation phenomenon of SBS C=C bonds under high temperatures and deep oxidation, leading to the formation of -OH, C=O and -COOH functional groups, which affect PMB viscosity [145]. According to these two hypotheses, a reactive rejuvenator should either act as a chain scission initiator or enable the SBS matrix to reverse the cross-linking after ageing.

**Figure 21:**
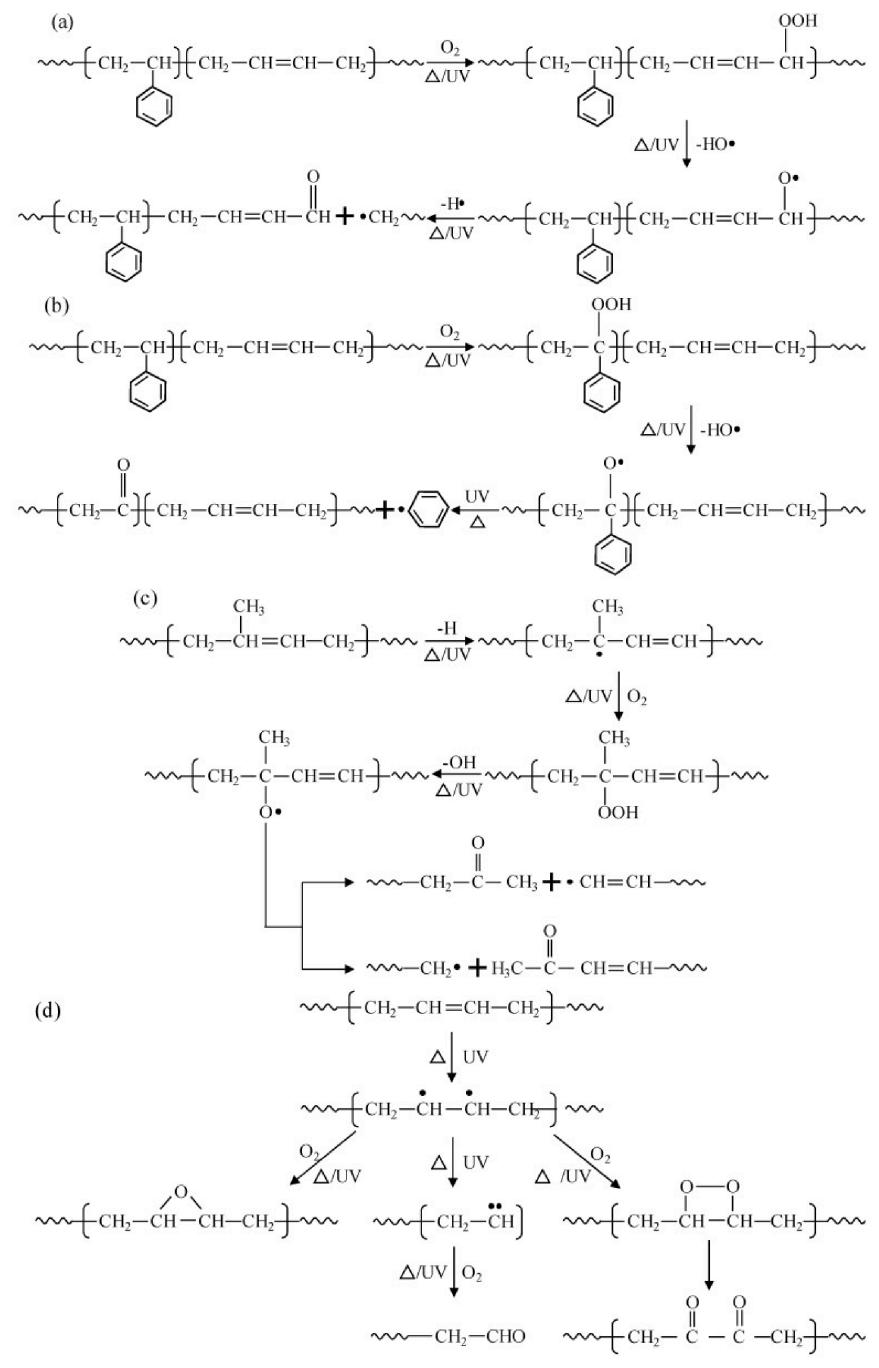
Reaction equation of degradation mechanism in polymer modifiers, (**a**) oxidation reactions of the α-H on the C=C double bond in styrene-butadiene copolymer, (**b**) oxidation reactions of the H on the benzene ring in styrene-butadiene copolymer, (**c**) oxidation reactions of the C=C double bond in natural rubber, (**d**) UV ageing with limited oxygen presence of the C=C double bond in PB generates a dicarbonyl compound, reprinted from [146] under the terms of open access (CC-BY 4.0 license).

It became crucial to determine the ageing factors causing each reaction. In 2026, Li L. et al. presented a study focused on SBS ageing reactions, presented in Figure 21 [146]. The study showed four reaction chains, three of them concerning SBS ageing ((a), (b) and (d)) and one describing reactions of natural rubber (c), of which an analysis is not included in this paper.

Reactions (a) and (b) occur under high oxygen concentration, where oxygen particles are bonding with either polybutadiene reactive allylic carbon ((a) CH_2_ on the right part of chemical formula) or polystyrene tertiary carbon ((b) the backbone carbon attached directly to the benzene ring), creating a -OOH hydroperoxide group. UV or/and heat cleaves the peroxide bond, leaving an alkoxy radical (-O*). The backbone of polymer in each pathway splits and creates a broken chain with a carbonyl group and reactive radical *CH_2_- (a) or benzene ring fracture (b). Between these reactions, polybutadiene is more prone to oxidation due to the weakly bound α-Hydrogen connected to the C=C bond. Both of these reactions are classified as the degradation process, since there is a chain scission of the polymer matrix to smaller particles.

However, reaction (d) shows what happens if the oxygen presence is limited. Without oxygen bonding after C=C cleavage, the double bond opens into an active diradical state (-CH*-CH*-). From there, three different reactions may occur—two under some oxygen presence (epoxidation, where polybutadiene forms a -CH-O-CH- stable epoxide ring or dioxetane cleavage, creating unstable 1,2-dioxetane and, consequently, chain scission; otherwise, polybutadiene degradation may occur). The last reaction, however, occurs with the least amount of oxygen, where under high temperatures or/and heat, the unpaired electrons on the activated carbon atoms search for stability. Without oxygen, the nearest compounds are other polymer chains with radical sites, creating a rigid matrix with a strong, permanent C-C bond. Since asphaltenes contain condensed polycyclic aromatic rings with C=C double bonds, although these bonds are far more stable than the C=C bond in the PB block; an aggressive polymer radical “attacks” the electron of the asphaltene ring, forcing its way into the structure and bridging the SBS fragment directly into the massive asphaltene cluster. This reaction becomes a compaction nightmare because of the rigid, brittle structure of the created bitumen. This is especially important in the consideration of mixing plants, where access to oxygen is particularly limited due to raw bitumen oxidation ageing.

Since excessive cross-linking (vulcanization) is irreversible, typical rejuvenation that also influences SBS polymer must focus on polymer degradation [147]. To reverse this ageing effect, compounds capable of reacting with the hydroxyl and carboxyl groups formed during chain scission and oxidation are required to reconnect SBS chains [148,149]. Xu et al. distinguish four types of Reactive Rejuvenators (RR) [150,151]:

- epoxy systems,
- isocyanate systems,
- compound systems,
- polymer systems.

**Figure 22:**
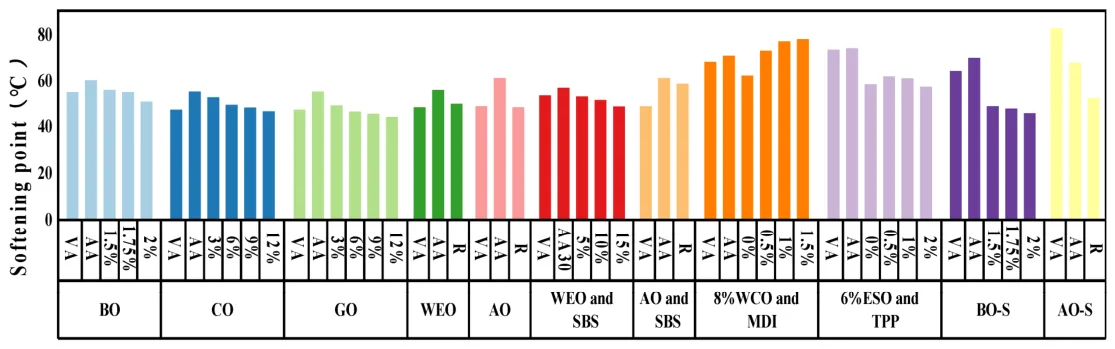
Softening point of virgin asphalt (VA), aged asphalt (AA) and rejuvenated asphalt (RA), reprinted from [150] under the terms of open access (CC-BY 4.0 license).

**Figure 23:**
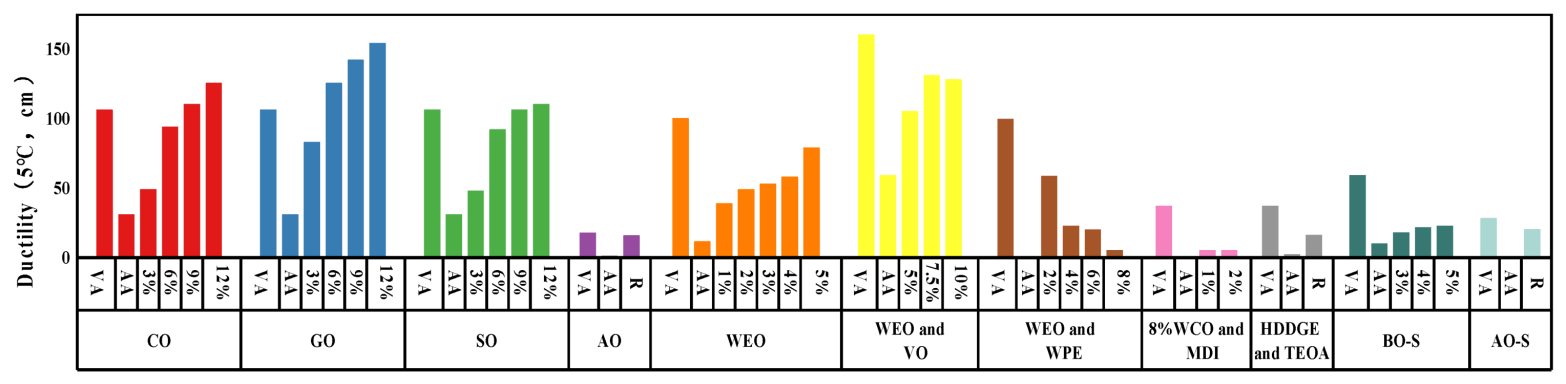
Ductility of virgin asphalt (VA), aged asphalt (AA), rejuvenated asphalt (RA) and waste polyethylene (WPE), reprinted from [150] under the terms of open access (CC-BY 4.0 license).

The epoxy system uses epoxides and a catalyst to reduce the energy required to recreate the polymer matrix, for example, epoxidized soybean oil (ESO) and Triphenylphosphine as catalyst (TPP) [152]. Alternative approaches use curing agents instead of catalysts. The difference between a catalyst and a curing agent is that a catalyst does not participate in the reaction itself but accelerates it, whereas a curing agent becomes a part of the cross-linked structure [153]. In contrast, isocyanate system does not need a catalyst. Examples include hexamethylene diisocyanate (HDI), diphenyl-methane-diisocyanate (MDI) and toluene-2, 6-diisocyanate (TDI) [154]). Generally, epoxy systems are proven to favor the recovery of low-temperature properties, while isocyanate systems favor high-temperature ones, recreating the SBS matrix by creating more flexible bonds, which translates into restoring rutting resistance and complex modulus (Figure 22 and Figure 23) [150].

Compound systems consist of both epoxides with catalysts and isocyanates. Polymer systems can be composed of various types of polymers (for example, reactive single component Polyurethane (PU) [155], hydroxyl terminated polybutadiene (HTPB) [156] and others [148]).

Polymer systems use not only small-molecule monomers but also large macromolecular chains that already possess active functional groups joining with the aged SBS. In case of cross-linked and gel-like bitumen, polymer structures diffuse aged colloidal system by adding light carrier oils and relatively “taking over” vulcanized polymer [156].

The effects of using RRs can be evaluated using various techniques, including spectroscopy and microscopy. However, the most reliable evidence of a restored cross-linked structure is the presence of a ‘plateau’ region in the temperature sweep curve obtained from DSR [148].

**Figure 24:**
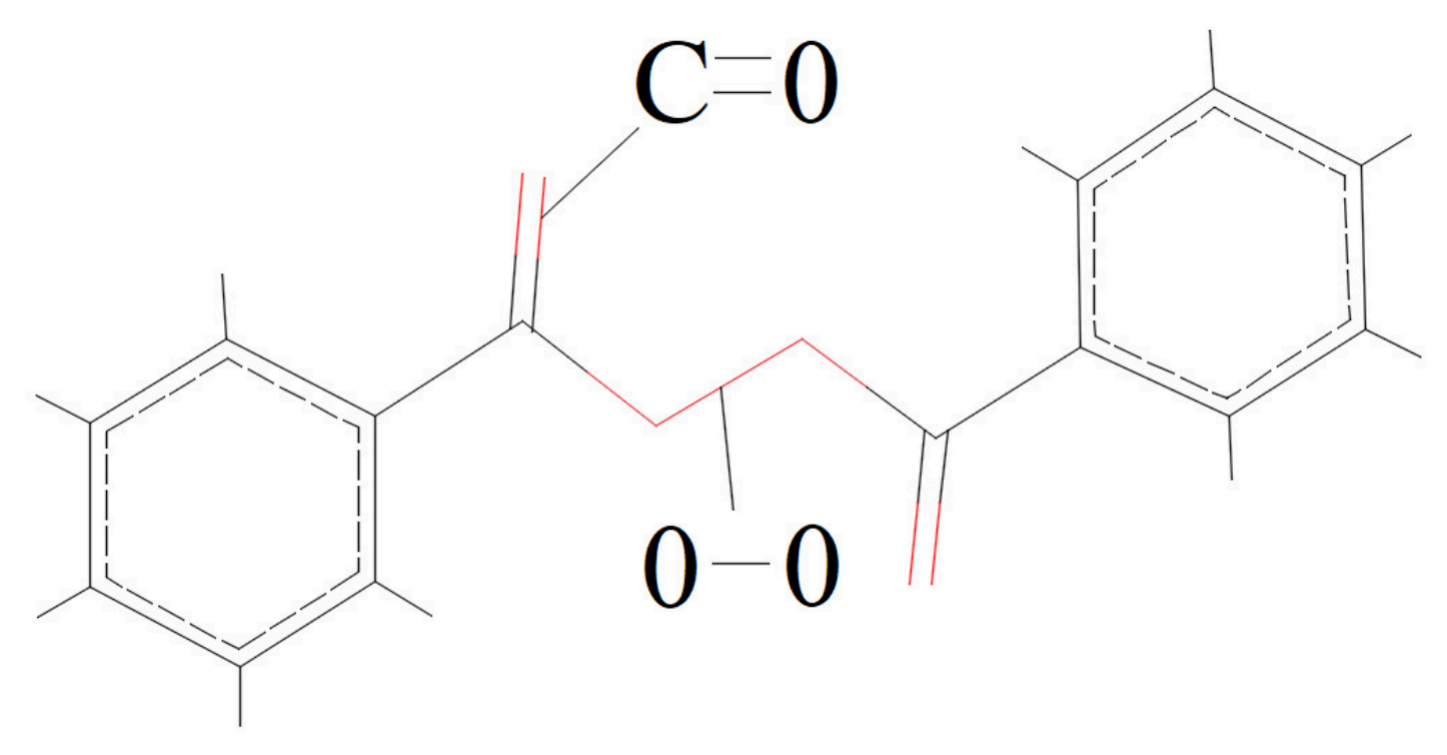
Dibenzoyl peroxide, reprinted from [157] under the terms of open access (CC-BY 4.0 license).

**Figure 25:**
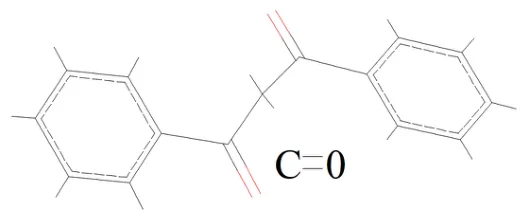
Dibenzoylmethane, reprinted from [157] under the terms of open access (CC-BY 4.0 license).

The only method to directly address excessive cross-linking is prevention, achieved by controlling the production temperature or using appropriate chemicals. One such proposals was presented in 2025, involving reactions with dibenzoyl peroxide (BPO (Figure 24)) and dibenzoylmethane (BPA (Figure 25)) [157]. Under the heat influence, BPO thermally decomposes into benzoic acid free radicals with strong oxidizing properties and high chemical reactivity. As these radicals open the SBS chains, the active carbonyl groups of BPA capture the free radicals, forming stable products and thereby preventing excessive cross-linking of the SBS polymer.

## 4. Conclusions

Styrene–Butadiene–Styrene (SBS)-modified bitumen effectively bridges the performance gap between conventional bitumens and high-curability pavement requirements. However, its complex chemical structure introduces significant challenges for long-term ageing prediction and recycling. Based on the comprehensive synthesis of current literature, the following global conclusions, industrial implications and future recommendations are established:

### 4.1. Structural Syntheses and Data Trends

- The Phase Inversion Threshold: The literature establishes a definitive cross-linking threshold at 5% to 8% SBS concentration. Below this range, the bitumen forms a matrix with dispersed polymer particles; above it (HiMA), the continuous polymer network dominates, shifting temperature sensitivity and creating a predictable mechanical plateau between 30 °C and 60 °C.
- Mathematical Modeling: Predicting PMB physical properties over varying dosages requires quadratic or cubic frameworks, depending on the evaluated parameters. Statistical evaluation proves that linear models fail to predict rheological parameters of bitumens, especially at higher concentrations of SBS.
- Chemical and Mechanical Divergence: Ageing mechanisms follow a two-stage kinetic pathway: rapid short-term sulfoxide group formation followed by long-term carbonyl formation. Mechanically, while conventional bitumen moderately links stiffness with fatigue strain after 10^6^ cycles of bending, SBS-modified mixtures exhibit a strong inverse relationship (r = 0.969), proving that excessive initial rigidity heavily penalizes long-term fatigue life.

### 4.2. Industrial and Environmental Applications

- RAP Processing Hazards: The common industrial practice of cold-dosing RAP with HiMA creates microstructural risks. When recycled material encounters superheated aggregates (up to 300 °C) in low-oxygen plant environments, the opened polybutadiene double bonds form permanent, rigid carbon bonds directly with heavy asphaltene clusters, leading to brittle mixtures and poor field compaction. This requires reevaluation of the levels of oxygen deprivation at mixing plant, where while oxygen rapidly ages bitumen, the lack of it almost entirely prevents effective use of HiMA RAP.
- Advanced Rejuvenation Pathways: Traditional soft bitumen acts only as physical volumetric spacers, often reducing rutting resistance. Industrial recycling of bitumens requires rejuvenators that chemically diffuse info SARA fractions, limiting the use of fresh bitumen, which cuts the costs of recycling. In case of HiMA, reactive rejuvenators are required to restore the oxygen-degraded polymer matrix. Low-oxygen cross-linking is irreversible and has to be prevented.
- Mandatory Inclusion of UV Radiation: Standard PAV ageing relies strictly on thermos-oxidation under pressure, completely omitting surface photolytic reactions. Because UV radiation dynamically cleaves polybutadiene and polystyrene bonds, it is recommended that future international standards introduce mandatory combined UV-thermal ageing protocols for upper pavement layers.

## Figures and Tables

**Table 1:** Residual Sum of Squares (RSS) comparison and error reduction matrix between the Linear and Quadratic ANCOVA frameworks based on data from [67].

Bitumen Physical Property	Linear Model RSS	Quadratic Model RSS	RSS Reduction [%]
Structural Viscosity	0.1308	0.0632	51.7
Softening Point	265.72	42.47	84.0
Penetration	361.53	152.86	57.7
Ductility	31.3	11.58	63.0

**Table 2:** Comparison of the average penetration, softening point and Fraass breaking point results for bitumen produced between 2018 and 2020 [86].

Binder Type		Penetration [0.1 mm]	Softening Point [°C]	Fraass Breaking Point [°C]
Raw binder	35/50	43.7	53.8	−12.6
	50/70	61.4	48.9	−14.8
	70/100	89.8	44.9	−16.2
HiMA	25/55-80	49.7	95.2	−21.2
	45/80-80	67.0	92.6	−20.9
	65/105-80	86.8	90.5	−21.2

**Table 3:** Summary of polynomial ANCOVA results for the complex modulus (logG*) of unmodified and polymer modified asphalt binders, based on data from [67].

Parameter/Source	1% β	3% β	5% β	1% SE	3% SE	5% SE
Intercept (LSBS baseline)	3.1407 ***	3.2174 ***	3.3158 ***	0.004	0.004	0.004
Temperature (Linear effect)	−10.2284 ***	−9.5447 ***	−8.7619 ***	0.031	0.033	0.034
Modifier Type: Unmodified matrix	−0.1955 ***	−0.2722 ***	−0.3707 ***	0.006	0.006	0.006
Modifier Type: RSBS polymer	−0.0128 *	0.0619 ***	0.2206 ***	0.006	0.006	0.006
Linear Temperature × Unmodified matrix	−0.4088 ***	−1.0926 ***	−1.8754 ***	0.044	0.047	0.047
Linear Temperature × RSBS polymer	0.0508 ns	0.2661 ***	0.7005 ***	0.044	0.047	0.047

***—*p* < 0.001; **—*p* < 0.01; *—*p* < 0.05; ns—non-significant.

**Table 4:** Summary of polynomial ANCOVA results for the rutting parameter (logG*/sin δ) of unmodified and polymer modified asphalt binders, based on data from [67].

Parameter/Source	1% β	3% β	5% β	1% SE	3% SE	5% SE
Intercept (LSBS baseline)	3.1473 ***	3.2292 ***	3.3411 ***	0.004	0.004	0.005
Temperature (Linear effect)	−10.2813 ***	−9.6153 ***	−8.834 ***	0.03	0.032	0.035
Modifier Type: Unmodified matrix	−0.1995 ***	−0.2815 ***	−0.3934 ***	0.006	0.006	0.007
Modifier Type: RSBS polymer	−0.0132 *	0.0679 ***	0.2447 ***	0.006	0.006	0.007
Linear Temperature × Unmodified matrix	−0.3835 ***	−1.0495 ***	−1.8308 ***	0.043	0.046	0.049
Linear Temperature × RSBS polymer	0.0518 ns	0.2705 ***	0.7736 ***	0.043	0.046	0.049

***—*p* < 0.001; **—*p* < 0.01; *—*p* < 0.05; ns—non-significant.

**Table 5:** ANCOVA test results based on data from [139].

Parameter/Source	ITS	ITSR	Resilient Modulus	Rutting Parameter	ITP
Intercept (Virgin base-line)	+2.291 ***	+83.162 ***	+6639.975 **	+4.448 ***	+0.591 ***
RAP Content (Linear/Quad effect)	+1.453 ***/−0.382 *	−0.189 **	+3065.827 */+443.793 ns	−0.027 ***	−0.008 ***
Rejuvenator: Soft Bitumen vs. Virgin	−0.924 **	+17.413 ***	−693.322 ns	−0.569 *	+0.221 **
Rejuvenator: Polanga oil vs. Virgin	−0.934 **	+13.758 ***	−1434.15 ns	+0.094 ns	+0.441 ***
RAP Content × Rejuvenator Interaction	-	−0.074 ns	-	−0.002 ns	+0.003 *

***—*p* < 0.001; **—*p* < 0.01; *—*p* < 0.05; ns—non-significant.

## Data Availability

The original contributions presented in this study are included in the article. Further inquiries can be directed to the corresponding author.
